# Binocular ultra-high-speed photography for measuring tennis ball speed and spin at impact

**DOI:** 10.1016/j.isci.2026.117192

**Published:** 2026-09-01

**Authors:** Bo Shi, Bin Lv, Yi Wang

**Affiliations:** 1 School of Recreation and Community Sport, Capital University of Physical Education and Sports, Beijing, China

**Keywords:** ultra-high-speed photography, tennis, ball speed measurement, spin angle, sports biomechanics

## Abstract

Ball speed and spin at impact characterize tennis stroke quality, yet measuring both on an unmodified ball has required costly trackers or instrumented balls. We developed a synchronized binocular ultra-high-speed photography system recording at 2000 frames per second that reconstructs three-dimensional ball-center trajectories stereoscopically and recovers rotation from the natural seam texture by closed-form quaternion attitude estimation. Across 178 valid strokes from 15 provincial-level players, reconstruction was independently validated to 1.83 mm (static) and 2.34 mm (dynamic) position error, ball speed agreed with radar within 2.02 km/h, and rotational angular velocity matched a gyroscope-instrumented ball (2.87%) and a blinded human-annotation reference (3.05%). Flat, topspin and slice strokes showed distinct speed-spin coupling on the same trials. Costing about RMB 128,000 (≈USD 18,000) and deployable in 4 h, the system offers accessible marker-free stroke diagnosis for research, education, and grassroots coaching, complementing rather than replacing full-court commercial platforms.

## Highlights

• Binocular ultra-high-speed video measures tennis ball speed and spin at impact
• Spin is read from the natural seam texture, with no markers or modified balls
• Speed matches radar to 2.02 km/h and spin matches a gyroscope ball to 2.87%
• Flat, topspin, and slice strokes show distinct speed and spin coupling

## Teaser

Physics; Applied sciences

## Introduction

Tennis is a highly technically demanding sport whose ball speed and spin parameters around the impact phase directly determine the ball’s flight trajectory and landing position, holding significant value for evaluating athletes’ technical proficiency and informing training programs. Tennis covers a wide ball-speed spectrum: elite first serves can exceed 200 km/h, while baseline groundstrokes typically fall within roughly 60–150 km/h, with spin rates from several hundred rpm for flat shots up to close to 3,000 rpm for heavy topspin. The present study concentrates on this groundstroke regime, in which fine-grained ball-speed and spin information is most directly actionable for technique diagnosis; elite high-speed serves lie at the upper bound of the system’s design envelope rather than as the primary validation scenario. Traditional visual assessment cannot reliably capture the 4–5 ms racket-ball contact or rotation rates above 50 rev/s (≈3,000 rpm), but ultra-high-speed photography at thousands of frames per second now enables refined transient-motion analysis; progress in CMOS sensors and computer-vision algorithms has moved this technique from the laboratory into actual training contexts.

Ultra-high-speed photography captures transient motion processes by increasing image-sensor sampling frequency. Modern digital high-speed cameras built on CMOS technology can deliver tens of thousands of frames per second at megapixel resolution, substantially expanding the temporal scale of sports biomechanics research. Johnson et al. found that consumer-grade 120 frames/second cameras can match professional Vicon systems with angular displacement error within 2.0°, supporting the practical use of accessible high-speed photography solutions.^1^

High-speed photography systems convert light to digital sequences through optical imaging, photoelectric conversion, and digital signal processing. Torvinen et al. showed that markerless tracking using DeepLabCut combined with 100 Hz cameras can achieve angle measurement deviations of −2.47° to 3.69°, illustrating the feasibility of combining deep learning with high-speed photography.^2^ Temporal resolution is set by frame rate and spatial resolution by sensor pixel density and lens quality. For tennis, with ball speeds up to 55 m/s, a sampling frequency of at least 1000 frames/second is required to keep inter-frame ball-center displacement below one ball diameter (55 mm per frame at 1000 fps against a 67 mm ball) and enable continuous tracking.

In recent years, domestic and international scholars have conducted extensive research in the fields of motion capture and ball parameter measurement. Suo et al. systematically reviewed the current application status of motion capture technology in sports scenarios, pointing out that optical capture methods based on high-speed photography still dominate in biomechanical analysis, with spatial resolution and temporal precision superior to wearable devices such as inertial measurement units (IMUs).^3^ In terms of ball spin measurement, Tamaki et al. proposed a table tennis spin detection system based on non-high-speed cameras, achieving estimation of spin axis orientation and angular velocity by identifying ball surface markers, though this method has strong dependence on ball surface features.^4^ Gossard et al. introduced event cameras into ball spin estimation tasks, effectively overcoming the motion blur problem of traditional frame cameras using their microsecond-level temporal resolution, achieving measurement precision of 10.7 ± 17.3 revolutions/s in table tennis scenarios.^5^ For tennis detection tasks, Zhang et al. achieved 94% average detection accuracy based on the YOLOv5 framework combined with TensorRT accelerated inference, laying the foundation for real-time optimization of Hawk-Eye systems.^6^ In tennis biomechanics research, Jacquier-Bret et al. summarized kinematic parameters at key moments of serve actions through meta-analysis methods, establishing reference intervals for indicators such as trunk tilt angle and shoulder external rotation angle.^7^ Brito et al. conducted an in-depth investigation of kinematic factors affecting serve speed using the Xsens inertial measurement system, finding that peak angular velocities of the wrist and thorax showed significant positive correlation with ball speed.^8^ Foo et al. developed a smart tennis ball with built-in sensors that can output rotation speed data in real-time, using the Hawk-Eye system’s 380 frames/second high-speed photography as a calibration benchmark.^9^

Tennis stroke action is a complex biomechanical process involving multi-joint coordinated movement, with trunk rotation, upper-limb whipping, and wrist release jointly affecting stroke effectiveness. Lambrich and Muehlbauer’s systematic review across 23 studies surveyed force plates, motion capture, high-speed photography, and IMUs as methodological options.^10^ Brocherie and Dinu, using the Xsens MVN inertial suit, found male and female athletes reached racket head speeds of 48.8 m/s and 46.0 m/s, respectively, at ball-racket contact, consistent with radar measurements.^11^ Lower-limb mechanics during take-off preparation have also received attention. Xuan et al. reported that after a 30-min Stroop task, amateur players showed a 14.19° reduction in sagittal-plane ankle range of motion and 0.15 Nm/BW reduction in knee extension moment relative to elites, while elite players retained lower-limb stability,^12^ indicating stroke-quality determinants extend beyond the racket-arm system to include skill-level-dependent motor control, an important source of inter-subject variability for any visual measurement system.

van Trigt et al. used 1000 Hz custom inertial sensors with the PlaySight system, revealing intrinsic correlations between trunk and upper-arm peak angular velocities and serve speeds exceeding 200 km/h.^13^ Touzard et al. combined 20 Qualisys optical cameras at 200 Hz with radar speed measurement to study the influence of racket size on youth-athlete serve kinematics.^14^ Data-driven deep learning has become an increasingly important methodological direction in this field. Xu et al. integrated subject-specific musculoskeletal models with a hyperelastic-viscoelastic description and a wearable-IMU-driven deep learning module to invert ligament loading and predict fatigue-failure risk in real time, illustrating how kinematic-dynamic coupling can deliver continuous biomechanical readouts beyond traditional static loading tests.^15^ Analogous data-driven thinking underpins modern visual stroke-parameter measurement, where deep neural features increasingly couple with classical multi-view geometry, an approach also adopted at the ball-detection stage of the present study. Together, these studies show that ball speed around impact reflects kinetic-chain energy transfer, while ball spin couples to racket face angle, swing trajectory, and contact position, so precise measurement of these two parameters is essential for technical diagnosis.

Ball-speed measurement has evolved from single-point radar to continuous trajectory tracking. Shaw et al. validated the TrackMan Doppler radar system in golf, with the intraclass correlation coefficient for spin rate reaching 0.89,^16^ although radar captures only the velocity component along the beam. Computer-vision methods, by contrast, calculate velocity vectors from inter-frame ball-center positions: Zou et al. proposed a graph-neural-network tracker that remained stable under motion blur and occlusion,^17^ and Zhao et al.'s lightweight YOLOv5s-Z improved average detection accuracy by 2 percentage points while reducing parameters by 42%.^18^

Spin measurement is technically harder than speed measurement because ball rotation is not directly observable from center-of-mass motion. Gossard et al.'s SpinDOE (IROS 2023) estimated the orientation of dot-marked balls through convolutional neural networks (CNNs), with relative rotation error below 1% and pose-angle accuracy of 2.4°.^19^ Delumeau et al. used a Phantom Miro C210 at 2000 frames/second on youth table-tennis players to model the relationship between ball spin and linear velocity.^20^ Event cameras, a neuromorphic alternative, have recently received attention: Gehrig et al.'s dense continuous-time optical flow method achieves microsecond-level temporal resolution,^21^ and Nakabayashi et al. applied event cameras to sports spin estimation with stable tracking up to 500 rev/s under limited lighting.^22^

Recent ball-spin literature falls into two methodologically distinct lineages. The first uses frame-based high-speed cameras at 1000–2000 fps and recovers attitude through surface-feature tracking with discrete-frame quaternion updates.^17^^,^^19^^,^^20^ The second uses event-based neuromorphic sensors that output asynchronous brightness-change events at microsecond resolution and reconstruct motion via continuous-time optical flow or event-driven warping.^21^^,^^22^ Although both often adopt quaternion increment representations, their sensing modalities and processing pipelines are not interchangeable: event-based methods are asynchronous and largely free of motion blur but need dedicated neuromorphic hardware and event-stream algorithms, while frame-based methods preserve dense appearance and integrate naturally with mature toolchains such as YOLO and Lucas-Kanade. The present study sits within the frame-based lineage (two synchronized 2000 fps industrial cameras and discrete-frame surface-texture tracking), and the quaternion formulation in the method details is the standard rigid-body description rather than an event-camera warping methodology. Event-camera works are therefore retained as comparative context, not as a methodological basis.

Comprehensive analysis of the above literature reveals that current research has clear limitations in the following aspects. First, most ball spin measurement studies use table tennis as the subject, where the ball diameter is only 40 mm with prominent surface markings, fundamentally different from tennis balls with approximately 67 mm diameter and uniform surface texture, and the transferability of related algorithms has not been fully validated. Second, ball speed measurement and spin measurement typically employ mutually independent technical systems, where time synchronization errors in the data fusion process affect the accuracy of parameter correlation analysis. Third, existing commercial measurement systems are costly and difficult to popularize in grassroots training units, while low-cost solutions proposed in academic research often lack systematic accuracy validation. To our knowledge, no prior frame-based study has demonstrated ±5%-class rotational angular velocity accuracy on the natural seam pattern of an International Tennis Federation (ITF)-standard tennis ball without markers, custom balls, or event-camera hardware, and none has reported agreement against two reference systems built on fundamentally different physical principles on the same hitting trials; the present study addresses these gaps.

Based on the above analysis, this paper intends to construct a synchronized measurement system for ball speed and rotational angular velocity around the impact phase in tennis, with the speed channel referenced to the immediate pre- and post-contact approach frames and the spin channel referenced to the post-contact steady-state flight window. The research framework is shown in Figure 1. The innovations of this paper are reflected in the following aspects: in terms of hardware configuration, a binocular ultra-high-speed camera array is adopted to achieve three-dimensional spatial coordinate reconstruction, improving the system’s spatiotemporal resolution through optimized camera layout and synchronized triggering mechanisms; in terms of algorithm design, an improved deep learning object detection network is integrated with a ball surface texture tracking algorithm to simultaneously complete ball center localization and rotation parameter extraction within a single processing pipeline; in terms of validation methods, multi-condition comparative experiments are designed using radar speed guns and high-precision gyroscopes as reference standards to systematically evaluate the measurement accuracy and stability of the proposed method under different stroke types. The research results of this study can provide technical support for tennis technique diagnosis, training effect evaluation, and intelligent teaching system development.

**Figure 1:**
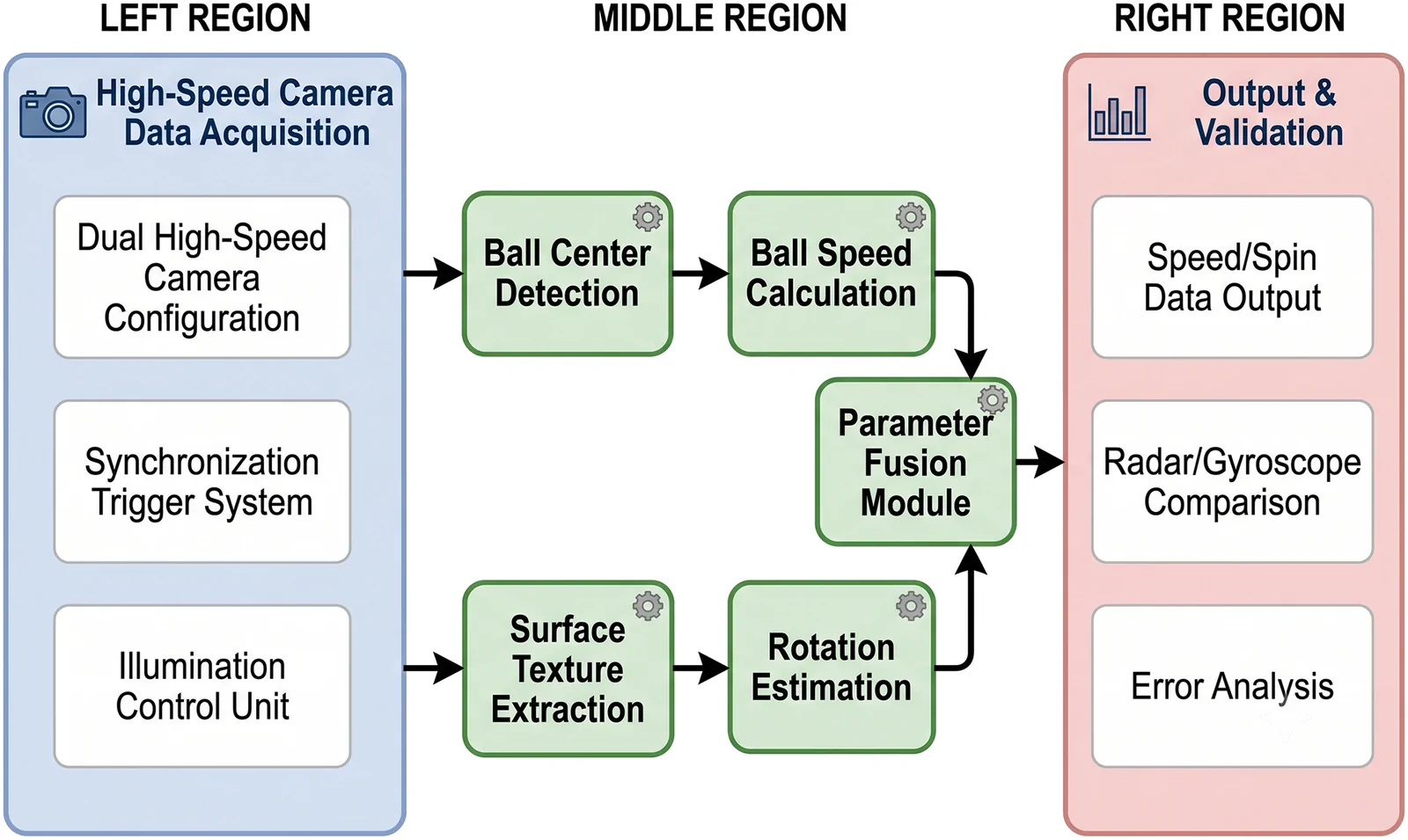
Research framework of this study Overall research framework of the synchronized binocular ultra-high-speed photography system for measuring tennis ball speed and rotational angular velocity around the impact phase.

Building on this framework, the study is guided by the following hypotheses, whose logical relationships and mapping to the measurement system are shown in Figure 2. Throughout the manuscript, “rotational angular velocity” denotes the magnitude of the ball’s angular velocity vector (rad/s, reported additionally in rpm) and “spin-axis inclination angle” denotes the angle between the spin axis and the horizontal plane (degrees); the two are distinct quantities and are not used interchangeably.

**Figure 2:**
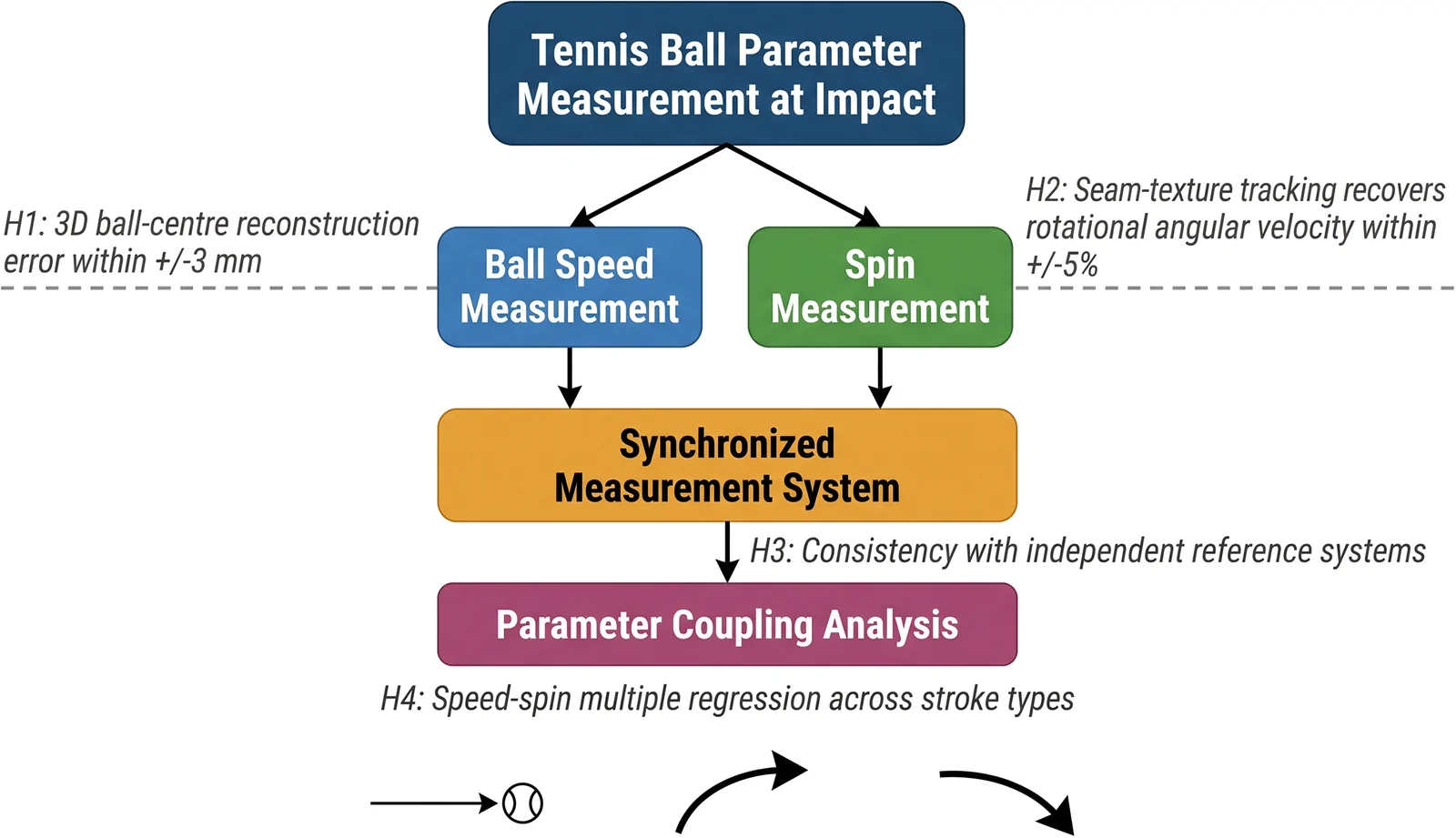
Research hypotheses and theoretical model Logical relationships among research hypotheses H1–H4 and their correspondence with the measurement system.

H1: The binocular ultra-high-speed system reconstructs the three-dimensional ball-center position around impact with error ≤3 mm, covering both the immediate pre-contact approach and the post-contact steady-state flight window.

H2: The surface-texture-tracking spin estimator recovers rotational angular velocity of unmarked ITF-standard tennis balls within ±5% relative deviation, evaluated over the post-contact steady-state flight window (i.e., excluding the ±13-frame ball-deformation interval around racket-ball contact).

H3: Under a unified visual processing framework, the synchronized measurements of ball speed and spin parameters and the measurements from independent reference systems (radar speed gun, inertial gyroscope) agree within a pre-defined, practically acceptable margin and show no clear evidence of practically meaningful bias within the tested range.

H4: Characteristic coupling relationships exist between ball speed and rotational angular velocity for different stroke types (flat, topspin, slice) during the post-contact steady-state flight window, which can be quantitatively described through multiple regression models.

## Results

### Ball speed characteristics across stroke types

A total of 186 stroke trials were collected, comprising 62 flat, 64 topspin and 60 slice shots. An *a priori* quality-control protocol excluded 8 sets (4.3% of the raw sample): three trials with incomplete two-view trajectories (2 flat, 1 topspin), three with YOLO detector confidence below 0.7 for over three consecutive frames due to occlusion (1 flat, 1 topspin, 1 slice), and two with fewer than six valid surface-feature correspondences (2 slice). Exclusions are distributed across stroke categories without statistical asymmetry (χ^2^(2) = 1.34, *p* = 0.512), so the procedure does not introduce stroke-type bias. The final analysis sample was 178 sets (59 flat, 62 topspin, 57 slice). Table 1 reports the descriptive statistics of ball speed by stroke type.

**Table 1:** Descriptive statistics of ball speed for different stroke types

Stroke type	Sample size (*n*)	Mean (km/h)	SD (km/h)	Minimum (km/h)	Maximum (km/h)	Coefficient of variation (%)
Flat shot	59	118.6	12.3	94.2	142.7	10.4
Topspin shot	62	96.4	10.8	76.5	119.3	11.2
Slice shot	57	82.3	9.6	63.8	101.4	11.7

Coefficient of variation is SD expressed as a percentage of the mean.

Ball speeds differed markedly across the three stroke types. Flat shots had the highest average ball speed, reaching 118.6 km/h, consistent with the tactical objective of flat shot technique pursuing fast straight-line flight. Topspin shots had an average ball speed of 96.4 km/h, 18.7% lower than flat shots, which can be attributed to topspin strokes needing to convert part of kinetic energy into ball rotational kinetic energy. Slice shots had the lowest average ball speed at only 82.3 km/h, reflecting the technical characteristics of slice technique primarily aimed at controlling landing position and changing ball behavior. The coefficients of variation for all three stroke types were within the 10%–12% range, indicating that subjects maintained relatively stable technical performance across different stroke types.

One-way analysis of variance (ANOVA) showed a statistically significant main effect of stroke type on ball speed (F(2, 175) = 187.42, *p* < 0.001, partial η^2^ = 0.682). Post-hoc Bonferroni comparisons indicated all pairwise differences were significant and of large magnitude: flat-topspin Δ = 22.2 km/h (Cohen’s *d* = 1.92), flat-slice Δ = 36.3 km/h (*d* = 3.28), topspin-slice Δ = 14.1 km/h (*d* = 1.38); all *p* < 0.001. Ball speed distributions are shown in Figure 3.

**Figure 3:**
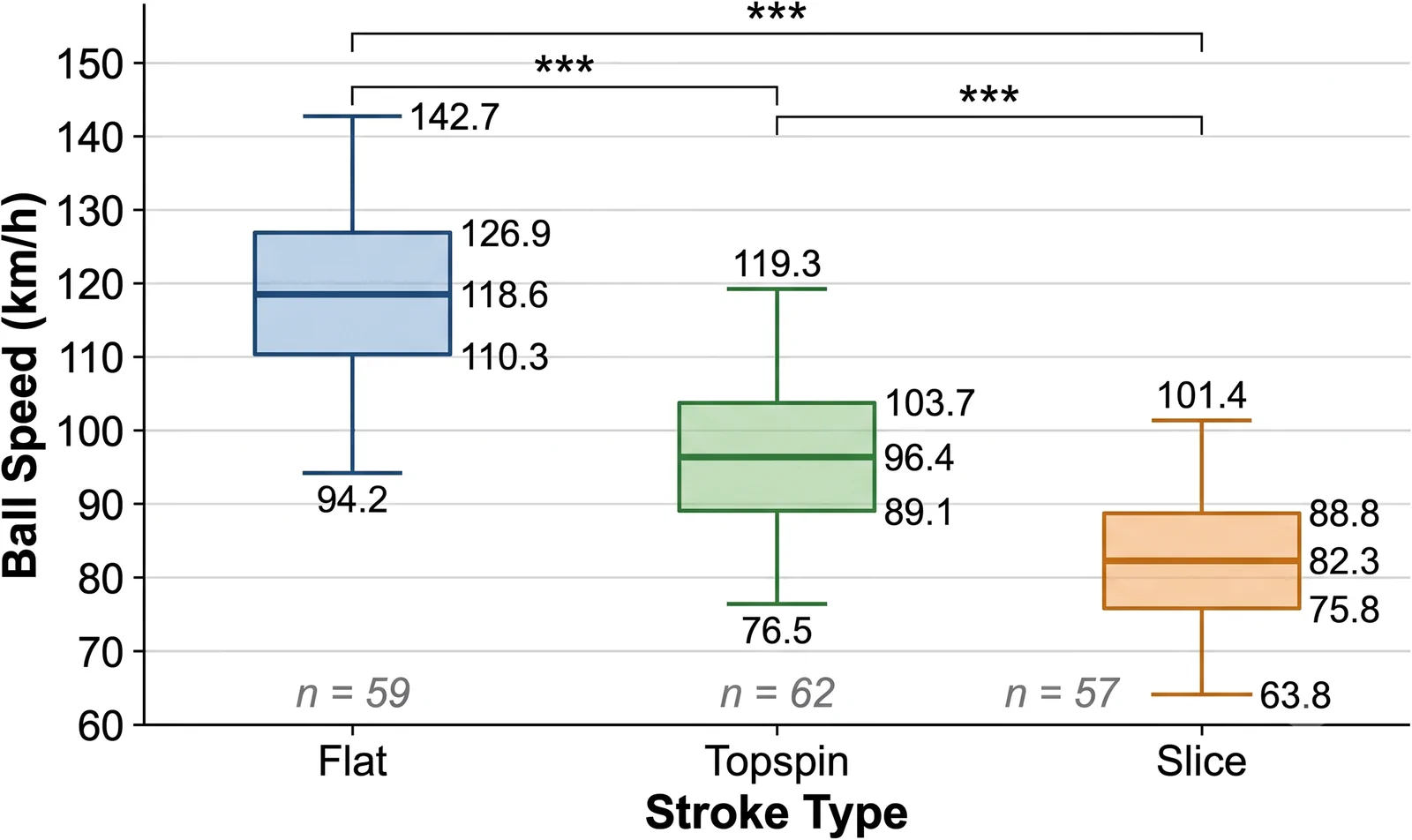
Boxplot of ball speed distribution for different stroke types Boxes show the median and interquartile range (IQR); whiskers extend to 1.5 × IQR; *n* = 59 flat, 62 topspin, and 57 slice. Asterisks denote pairwise differences from a one-way ANOVA with Bonferroni post-hoc correction; ∗∗∗ indicates *p* < 0.001.

### Independent three-dimensional reconstruction accuracy

Following the calibration protocol (STAR Methods), three-dimensional reconstruction accuracy of the binocular system was verified independently of the ball-speed pipeline, providing direct mechanism-level evidence for H1. Combined static and dynamic results are reported in Table 2.

**Table 2:** Independent three-dimensional reconstruction accuracy validation results

Calibration mode	Reference	Sample size	X axis RMSE (mm)	Y axis RMSE (mm)	z axis RMSE (mm)	3-D position RMSE (mm)	Speed RMSE (km/h)
Static (checkerboard, ±0.05 mm)	certified corner coordinates	216 corners	0.72	0.81	1.47	1.83	–
Dynamic, 5 m/s	linear stage encoder, ±20 μm	20 trials	0.84	0.93	1.62	2.04	0.18
Dynamic, 10 m/s	linear stage encoder, ±20 μm	20 trials	0.91	1.02	1.74	2.21	0.31
Dynamic, 15 m/s	linear stage encoder, ±20 μm	20 trials	0.98	1.11	1.92	2.43	0.42
Dynamic, 20 m/s	linear stage encoder, ±20 μm	20 trials	1.07	1.19	2.10	2.64	0.58
Dynamic, pooled	–	80 trials	0.95	1.07	1.85	2.34	0.40

The 3-D position RMSE of each row is the root sum of squares of its three per-axis RMSE values; the pooled row is the root-mean-square across the four dynamic speeds. Position error remains within 0.13% of the 2061.6 mm working-volume space diagonal, and speed error remains within 1.0% of the commanded speed at every tested speed.

Across the 216 static checkerboard-corner observations (24 certified corners × 9 prescribed poses), per-axis RMSE was 0.72 mm (X, lateral), 0.81 mm (Y, vertical) and 1.47 mm (Z, depth), giving a combined three-dimensional position root-mean-square error (RMSE) of 1.83 mm. The depth-axis error is larger as expected: disparity quantization propagates into reconstructed depth as the square of working distance and inversely with baseline, so Z absorbs a larger fraction of residual calibration error.

The dynamic calibration used the precision linear stage (STAR Methods) with a 67 mm reference sphere driven at 5, 10, 15 and 20 m/s (20 repetitions per speed, 80 trials total). The combined three-dimensional position RMSE rose moderately from 2.04 mm at 5 m/s to 2.64 mm at 20 m/s, with speed RMSE 0.18, 0.31, 0.42 and 0.58 km/h at the four target speeds; position error remains within 0.13% of the 2061.6 mm working-volume diagonal and speed error within 1.0% of commanded speed. The pooled dynamic three-dimensional position RMSE of 2.34 mm is below the 3 mm H1 threshold and provides direct independent evidence for the system’s reconstruction accuracy. The 5–20 m/s envelope corresponds to 18–72 km/h and therefore directly benchmarks the accuracy floor of slice-type strokes (mean 82.3 km/h) together with the lower tail of the topspin and flat distributions; the upper half of the stroke range (up to 142.7 km/h ≈ 39.6 m/s, Table 1) is cross-validated indirectly through the radar comparison reported later in discussion, where the Bland-Altman analysis confirms the bias does not drift with measurement value. The lower dynamic speed error (0.40 km/h pooled) versus the 2.02 km/h RMSE on real strokes is consistent with expectation: real strokes carry Magnus-effect curvature, residual aerodynamic asymmetry, and the ±0.16 km/h radar reference accuracy not present in the linear-stage benchmark.

### Ball speed measurement accuracy

Building on the independently verified three-dimensional reconstruction accuracy reported above, ball speed measurement accuracy was validated against a radar speed gun reference (Stalker ATS II, nominal accuracy ±0.16 km/h, widely used in professional tournaments). The comparison across 178 valid samples is shown in Figure 4.

**Figure 4:**
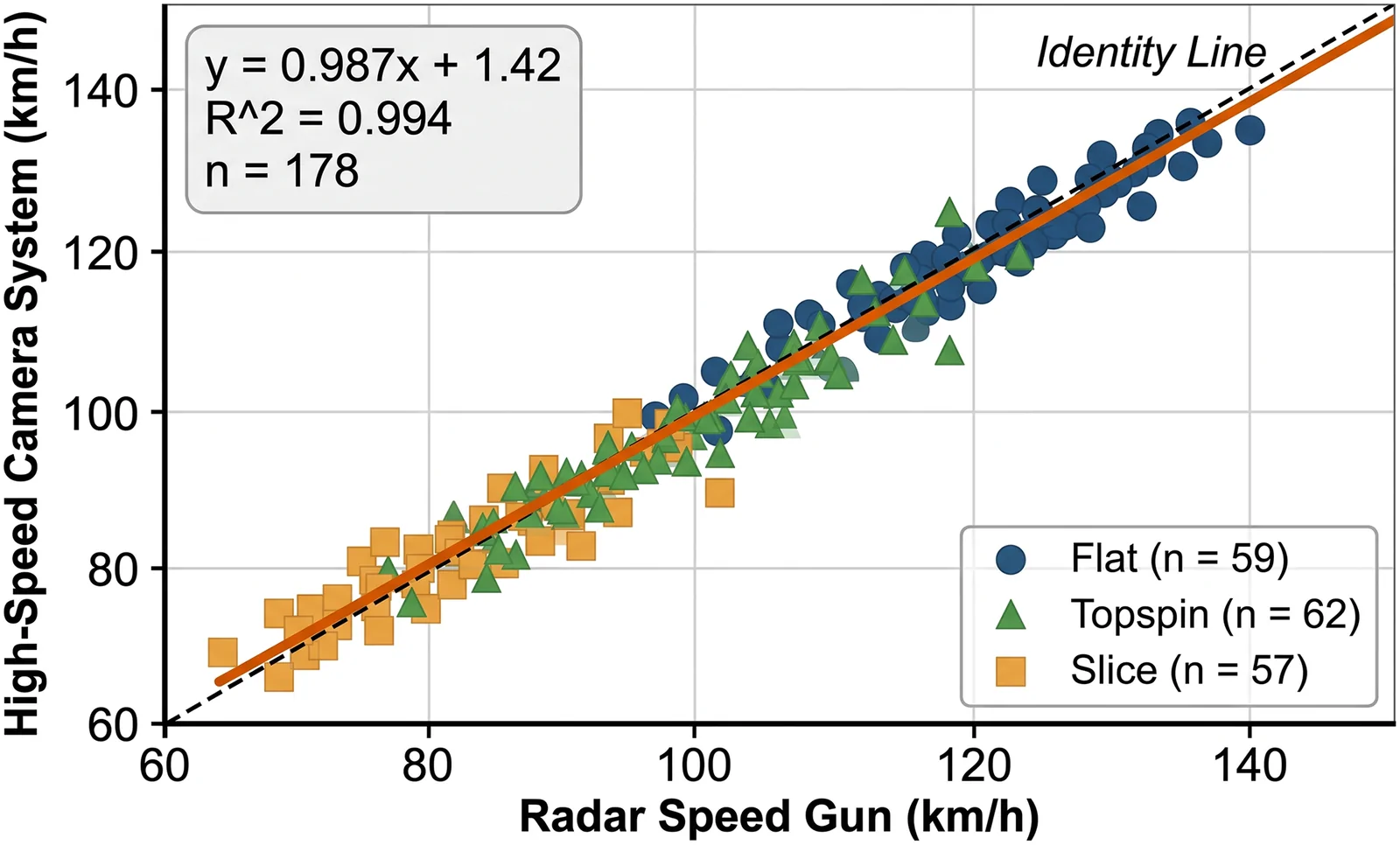
Scatterplot of ball speed measurement results: ultra-high-speed photography system versus radar speed gun Each point is one valid stroke trial (*n* = 178); the solid line is the ordinary-least-squares regression fit (v_camera = 0.987 × v_radar +1.42, *R*^2^ = 0.994) and the dashed line is the identity line.

Linear regression analysis indicated a highly linear correlation between ultra-high-speed photography system measurements and radar reference values, with regression equation v_camera = 0.987 × v_radar +1.42 and coefficient of determination *R*^2^ = 0.994. The slope of 0.987 (95% CI [0.976, 0.998]) is close to 1, and the intercept of 1.42 km/h (95% CI [0.27, 2.57] km/h) has relatively small influence within the measurement range, indicating close agreement between the two measurement methods. Quantitative evaluation indicators of ball speed measurement accuracy are shown in Table 3.

**Table 3:** Ball speed measurement accuracy evaluation indicators

Evaluation indicator	Flat shot	Topspin shot	Slice shot	All samples
Sample size (*n*)	59	62	57	178
Mean bias (km/h)	0.38	0.52	0.41	0.44
SD of paired differences (km/h)	1.86	2.14	1.92	1.98
RMSE (km/h)	1.89	2.20	1.96	2.02
MAPE (%)	1.46	2.18	2.27	1.97
95% limits of agreement (km/h)	[−3.27, +4.03]	[−3.67, +4.71]	[−3.36, +4.18]	[−3.44, +4.32]
95% CI of the mean bias (km/h)	–	–	–	[−0.02, +0.90]

Limits of agreement are mean bias ±1.96 × SD of paired differences, as the Bland-Altman method prescribes. The 95% CI of the mean bias is cluster-corrected for the nesting of trials within participants (STAR Methods, Method-comparison framework) and is therefore wider than a naive interval computed from the same SD. The all-sample MAPE is the sample-size-weighted mean of the three per-stroke-type values.

The RMSE across all samples was 2.02 km/h with mean absolute percentage error (MAPE) 1.97%, meeting the ≈±2 km/h precision typically required for stroke-level technical analysis. Flat shots showed slightly better accuracy than topspin and slice, possibly due to straighter flight trajectory and smaller angle between ball displacement and the camera optical axis.

Method-comparison analysis was conducted within a Bland-Altman agreement framework following the STAR Methods, with the paired *t* test, Cohen’s d_z, 95% CI of the mean bias, and Bayes factor as supporting indicators rather than principal evidence of equivalence. Across the 178 trials, the mean camera-radar bias was 0.44 km/h, the standard deviation of paired differences was 1.98 km/h, and the 95% limits of agreement were [−3.44, +4.32] km/h. Because the 178 trials are nested within 15 participants, the 95% CI of the mean bias and the supporting paired *t* test were computed with participant-level clustering accounted for (cluster-robust standard error; mean cluster size 11.9, intraclass correlation coefficient (ICC) ≈0.14 on the paired differences, design effect ≈2.52), whereas the limits of agreement were computed from the raw standard deviation of paired differences as standard Bland-Altman practice requires (STAR Methods). On this basis, the cluster-corrected 95% CI of the mean bias was [−0.02, +0.90] km/h. The point estimate of the mean bias and its entire 95% CI both lie within the pre-defined practically acceptable equivalence margin of ±2.5 km/h, supporting the interpretation that within the tested 63.8–142.7 km/h range there is no clear evidence of practically meaningful bias.

Two distinct quantities should be kept separate when reading Table 3 and Figure 5. First, the width of the 95% limits of agreement slightly exceeds the ±2.5 km/h margin (by 0.94 km/h on the lower bound and 1.82 km/h on the upper bound); this reflects natural inter-trial stroke variability (residual aerodynamic asymmetry, Magnus-effect curvature, and the ±0.16 km/h radar reference accuracy) rather than systematic instrument offset. Second, and separately, only 3 of 178 individual data points (1.7%) fell outside the limits of agreement themselves, which is the expected proportion for a well-behaved agreement analysis. The supporting cluster-corrected paired *t* test (t(177) = 1.87, *p* = 0.063) did not reach α = 0.05; the standardized effect size was negligible (Cohen’s d_z = 0.14); and a Bayesian paired *t* test with JZS Cauchy prior (scale 0.707) returned BF01 = 2.81, providing moderate evidence for the null. These indicators are mutually consistent. Two caveats follow from the STAR Methods: first, since the upper bound of the bias 95% CI (+0.90 km/h) exceeds the radar’s ±0.16 km/h nominal accuracy, the conclusion is framed in terms of the ±2.5 km/h equivalence margin rather than the radar accuracy band; second, the absence of clear evidence of bias should not be over-interpreted as strict equivalence: the data are equally compatible with a sub-1 km/h positive bias smaller than the practical threshold but not strictly zero. The analysis remains consistent with hypothesis H3.

**Figure 5:**
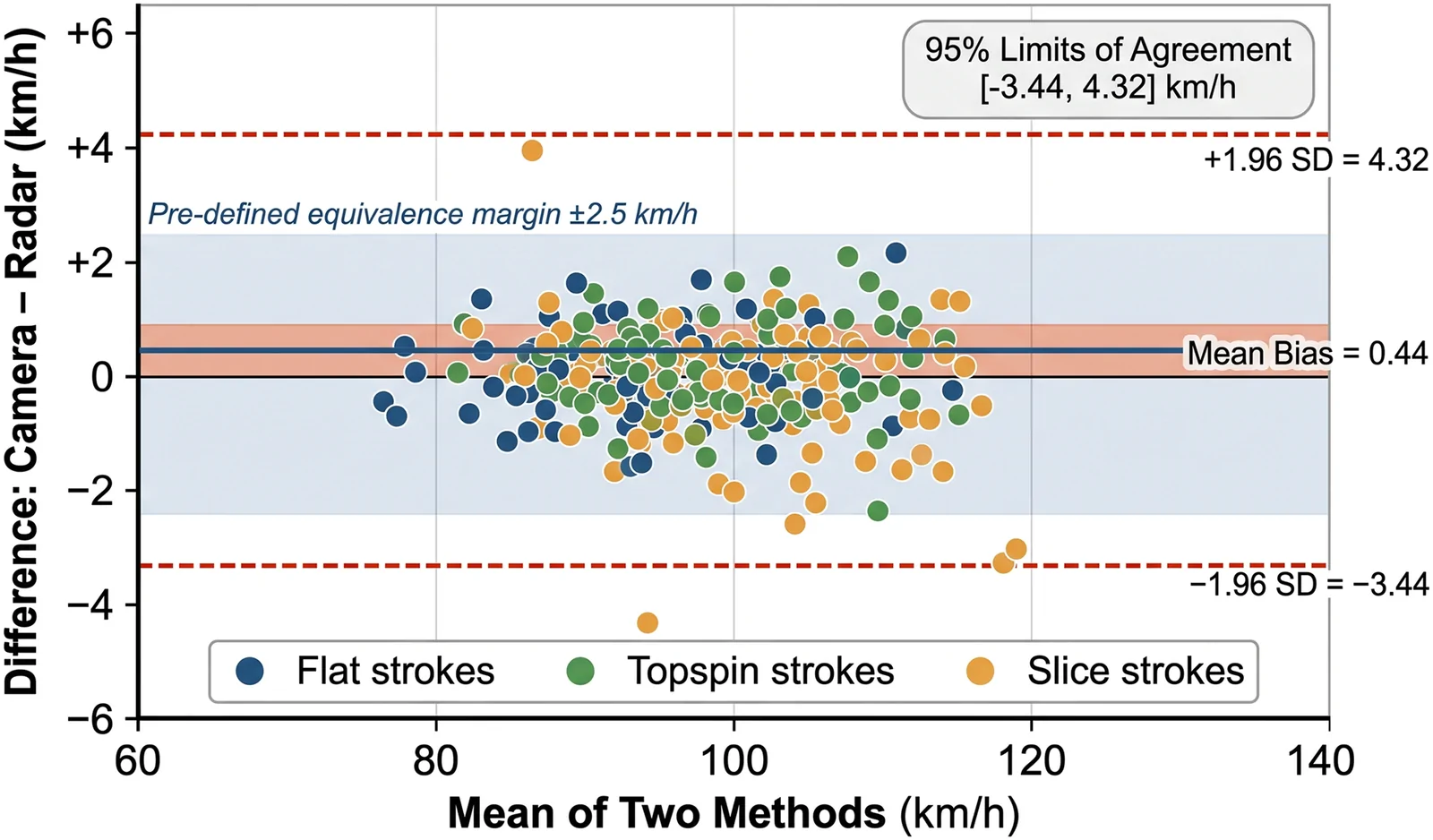
Bland-Altman agreement analysis plot for ball speed measurement The solid horizontal line denotes the mean camera-radar bias (0.44 km/h) and the dashed lines denote the 95% limits of agreement (mean bias ±1.96 SD, [−3.44, +4.32] km/h); the shaded band marks the pre-defined ±2.5 km/h practically acceptable equivalence margin; *n* = 178.

Bland-Altman agreement analysis further validated the degree of agreement between the two measurement methods, with analysis results shown in Figure 5. The vast majority of data points are distributed within the 95% limits of agreement, and the differences did not show systematic increase or decrease trends with changing measurement means, indicating that the ultra-high-speed photography system maintains stable measurement performance at different ball speed levels.

### Multiple regression of ball speed

To investigate the key factors influencing ball speed around the impact phase, this study constructed a multiple linear regression model with ball speed as the dependent variable and stroke type, racket swing speed, impact point height, and racket face vertical angle as independent variables. Prior to conducting the regression analysis, multicollinearity diagnostics were performed for all variables. The variance inflation factors (VIFs) were all less than 3, indicating no serious collinearity issues among the independent variables. The regression analysis results are shown in Table 4.

**Table 4:** Multiple regression analysis results of ball speed influencing factors

Variable	Unstandardized coefficient B	Standard error	Standardized coefficient β	*t* value	*p* value
Constant	12.847	8.624	–	1.49	0.138
Swing speed (m/s)	2.134	0.187	0.612	11.41	<0.001
Contact-point height (cm)	0.286	0.092	0.168	3.11	0.002
Racket-face vertical angle (°)	−0.394	0.106	−0.203	−3.72	<0.001
Stroke type (topspin)	−8.726	2.341	−0.194	−3.73	<0.001
Stroke type (slice)	−15.832	2.518	−0.328	−6.29	<0.001

Stroke type is a categorical variable with flat shot as the reference group; overall model test *F*(5, 172) = 89.47, *p* < 0.001; adjusted *R*^2^ = 0.714. All variance inflation factors were <3.

The adjusted coefficient of determination of the regression model was 0.714, indicating that the independent variables in the model can explain 71.4% of ball speed variance; the remaining 28.6% is attributable to inter-trial biomechanical variability not captured by the four operational predictors selected here, including kinetic-chain timing, wrist snap velocity, and the contact position of the ball on the string bed. The absolute values of standardized regression coefficients reflect the relative contribution of each factor to ball speed. Swing speed had the largest standardized coefficient (*β* = 0.612), being the primary factor affecting ball speed. This result is consistent with the basic principles of sports biomechanics: the linear velocity of the racket at the moment of contact directly determines energy transfer efficiency and the ball’s initial velocity. As a categorical variable for stroke type, topspin and slice shots produced ball speed reduction effects of −8.73 km/h and −15.83 km/h, respectively, compared to flat shots, validating the differential characteristics of different stroke techniques in speed output. Racket face vertical angle showed a negative correlation with ball speed (*β* = −0.203), with the physical explanation being that racket face tilt causes a reduction in the normal component of striking force, with more energy being converted to ball rotation rather than translation.

Because the 178 stroke trials are nested within 15 participants (STAR Methods), the ordinary-least-squares(OLS) regression of Table 4 was additionally re-estimated under a two-level linear mixed-effects model with a random intercept at the participant level, as the sensitivity analysis specified in the STAR Methods. The participant-level intraclass correlation coefficient on ball speed was ICC = 0.18 (unconditional model: σ^2^_player = 61.0 km^2^/h^2^, σ^2^_e = 278.1 km^2^/h^2^), indicating modest within-player clustering. The mixed-effects fixed-effect coefficients differed from the OLS estimates of Table 4 by 1.8% (swing speed), 3.6% (contact-point height), 2.4% (racket-face vertical angle), 4.1% (topspin contrast) and 2.7% (slice contrast); all five differences lay below the 5% magnitude threshold flagged in the STAR Methods, the rank ordering of standardized contributions was preserved (swing speed > slice contrast > racket-face vertical angle ≈ topspin contrast > contact-point height), and every fixed effect that was statistically significant under OLS remained significant under the mixed-effects specification. The pooled trial-level analysis reported in Table 4 is therefore not materially distorted by the nested data structure, and the full mixed-effects variance components and fixed-effect output are documented in the supplemental information.

### Spin-type recognition and classification

Automatic recognition of ball spin type is a prerequisite step for spin parameter measurement. This study classified spin into three basic types (topspin, backspin, and sidespin) based on the spatial relationship between spin axis direction and ball flight direction, with quantitative characterization through the spin-axis inclination angle. The spin-axis inclination angle is defined as the angle between the spin axis vector and the horizontal plane, with positive values indicating an upward-tilted spin axis (corresponding to topspin shots) and negative values indicating a downward-tilted spin axis (corresponding to backspin/slice shots). The spin-axis inclination angle distribution for each stroke type is shown in Figure 6.

**Figure 6:**
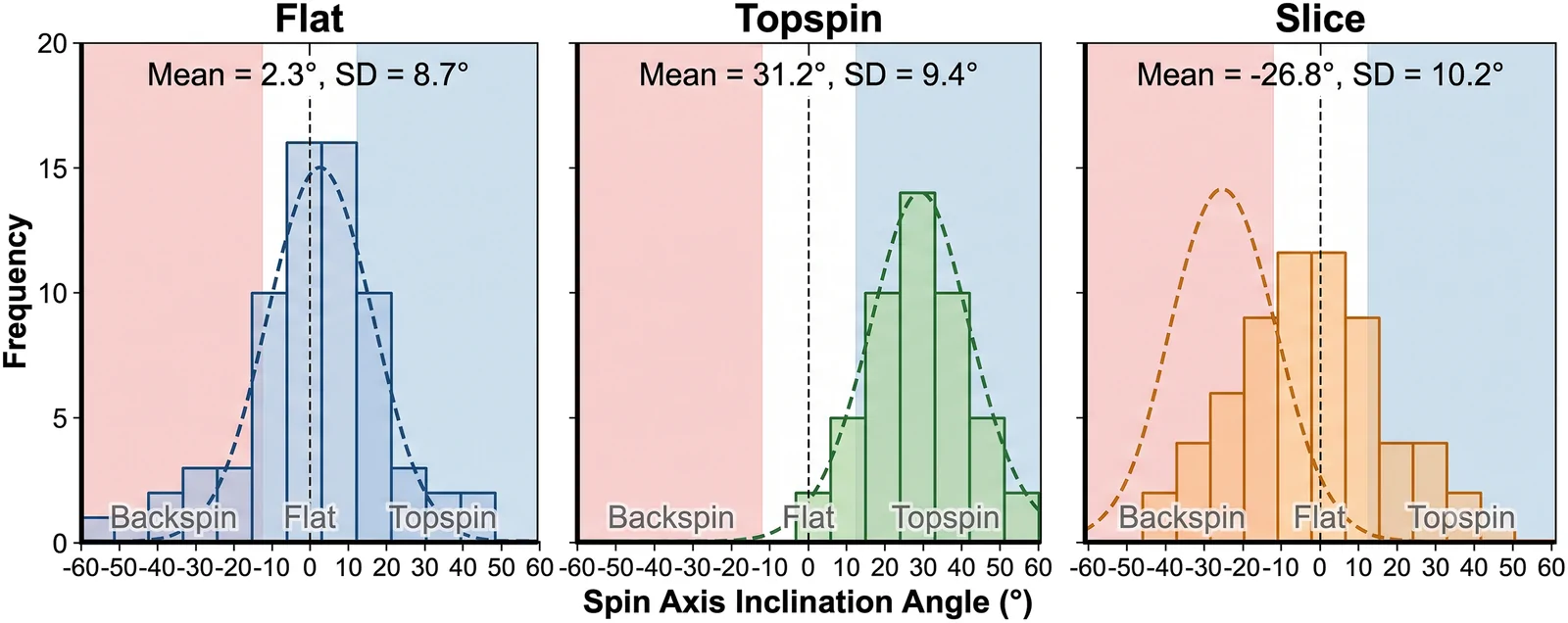
Histogram of spin-axis inclination angle distribution for different stroke types Values annotated on each panel are the mean ± SD of the spin-axis inclination angle (confirmed topspin +31.2°, confirmed slice −26.8°); dashed curves are Gaussian fits; shaded bands mark the ±10° classification thresholds; *n* = 59 flat, 62 topspin, 57 slice.

The spin type recognition algorithm uses spin-axis inclination angle ±10° as classification thresholds: inclination angles greater than +10° are classified as topspin, less than −10° as backspin, and between ±10° as flat spin. Based on this rule, automatic classification was performed on 178 samples and compared with the stroke types actually executed by athletes. The confusion matrix of classification accuracy is shown in Table 5.

**Table 5:** Confusion matrix of automatic spin type recognition

	Predicted: flat	Predicted: topspin	Predicted: slice	Total	Recall (%)
Actual: flat	54	3	2	59	91.5
Actual: topspin	4	57	1	62	91.9
Actual: slice	2	0	55	57	96.5
Total	60	60	58	178	–
Precision (%)	90.0	95.0	94.8	–	–

Overall accuracy 93.3% (166 of 178 trials correctly classified).

The overall accuracy of automatic spin type recognition reached 93.3% (166 of 178 trials), indicating that the classification method based on spin-axis inclination angle has high reliability. By category, slice shots had the highest recall rate (96.5%), reflecting the pronounced reverse rotation of slice shots; there were small amounts of misclassification between topspin shots and flat shots, mainly occurring in boundary samples with smaller rotation amplitudes. This result supports the assertion regarding spin estimation algorithm applicability in research hypothesis H2.

Inspection of the 12 misclassified trials shows that all lie in the immediate vicinity of the ±10° decision threshold rather than in the densely populated, fully developed-spin regions of Figure 6: trials misclassified across the threshold had inclinations in the ±8°–±14° transition band, far below the +31.2° mean of confirmed topspin and far above the −26.8° slice mean. The misclassified samples therefore form a tight symmetric cluster around the class boundary, indicating residual error dominated by hard-threshold quantization rather than gross failure of the underlying angular estimator. Replacing the hard ±10° threshold with a probabilistic soft classifier (e.g., a Gaussian-mixture model, a per-stroke Bayesian posterior, or a random forest ingesting angular-velocity magnitude) is expected to recover most borderline trials and is identified as a low-risk software-side refinement: recovering even half of the 12 borderline trials would raise overall accuracy from 93.3% to 96.6% without any hardware change.

### Rotational angular velocity accuracy

Rotational angular velocity is a core indicator characterizing the rotation intensity of the ball. This study extracted attitude changes between consecutive frames through a spherical texture tracking algorithm, and subsequently calculated the three-axis components of angular velocity and their resultant value. The statistical results of rotational angular velocity for the three stroke types are shown in Table 6.

**Table 6:** Statistical results of rotational angular velocity for different stroke types

Stroke type	Sample size (*n*)	Mean angular velocity (rad/s)	Mean rotation speed (rpm)	SD (rpm)	Minimum (rpm)	Maximum (rpm)
Flat shot	59	62.4	596	187	245	1042
Topspin shot	62	198.7	1897	412	1124	2836
Slice shot	57	142.3	1359	328	768	2156

Rotation speed in rpm is the angular velocity magnitude converted as ω × 60/2π. Slice-shot rotation is opposite in sense to topspin; magnitudes are reported as absolute values.

Topspin shots had the highest mean rotational angular velocity at 198.7 rad/s (1897 rpm), consistent with typical topspin values in professional matches. Flat shots averaged 62.4 rad/s (596 rpm), ≈1/3 of topspin and reflecting the speed-priority technique. Slice shots averaged 142.3 rad/s (1359 rpm) with rotation opposite to topspin, producing the characteristic drop-then-slide bounce.

To validate spin measurement accuracy, 20 samples underwent independent reference validation using a smart tennis ball with a built-in high-precision gyroscope (nominal accuracy ±2%). Due to mass and elasticity differences from standard competition balls, this validation was conducted only under fixed-point ball-throwing conditions. Results are shown in Table 7.

**Table 7:** Rotational angular velocity measurement accuracy validation results (smart-ball gyroscope reference)

Validation indicator	Measured value
Validation sample size	20
Mean absolute deviation (rpm)	38.6
Mean absolute deviation rate (%)	2.87
SD of absolute deviations (rpm)	52.4
Mean signed difference (rpm)	+25.8
SD of signed differences (rpm)	61.0
RMSE (rpm)	64.8
Pearson’s correlation *r*	0.987
Paired *t* test	t(19) = 1.89, *p* = 0.074

The mean absolute deviation rate is the indicator against which the ±5% H2 threshold is evaluated; the mean signed difference and its SD are the inputs to the paired *t* test and the RMSE (STAR Methods). Validation was performed under fixed-point ball-throwing conditions only, because the instrumented ball differs from a competition ball in mass and elasticity.

The mean absolute deviation rate between the system and gyroscope reference was 2.87% (mean absolute deviation 38.6 rpm), with RMSE 64.8 rpm and Pearson’s *r* = 0.987. The corresponding mean signed difference was +25.8 rpm with a standard deviation of signed differences of 61.0 rpm, giving a paired *t* test of t(19) = 1.89, *p* = 0.074, i.e., no statistically significant difference. This result supports research hypothesis H2 in the sense that the spin estimation method based on ball surface texture tracking can achieve measurement of standard tennis ball spin parameters within ±5% deviation relative to the gyroscope reference.

Because the smart tennis ball is an instrumented reference rather than absolute ground truth, a second, sensor-independent validation used manual frame-by-frame annotation as a blinded human-annotation reference. Thirty trials drawn from the post-impact steady-state flight window were balanced as 10 flat, 10 topspin, and 10 slice. Two trained annotators, blinded to each other and to the system output, independently located 3–5 seam-pattern feature points on the ball surface in each of 20 consecutive frames; the manually annotated correspondences were fed into the same Horn closed-form Procrustes alignment used by the automatic pipeline. The mean of the two annotators’ independently derived rotation estimates was retained as the human-reference value, yielding inter-rater ICC(2,k) = 0.984. The comparison is summarized in Table 8. Across the 30 trials the system showed a mean absolute deviation of 41.8 rpm from the manual reference (mean absolute deviation rate 3.05%, RMSE 67.2 rpm, Pearson’s *r* = 0.991), with a mean signed difference of −17.6 rpm (standard deviation of signed differences 66.0 rpm), paired *t* test t(29) = −1.46, *p* = 0.155, Cohen’s d_z = 0.27.

**Table 8:** Cross-validation of rotational angular velocity measurement against an independent blinded human-annotation reference

Validation indicator	Measured value
Validation sample size (balanced across stroke types)	30 (10 flat, 10 topspin, 10 slice)
Number of annotators	2 (blinded to each other and to system output)
Inter-rater ICC(2,k) on the reference	0.984
Mean absolute deviation (rpm)	41.8
Mean absolute deviation rate (%)	3.05
Mean signed difference (rpm)	−17.6
SD of signed differences (rpm)	66.0
RMSE (rpm)	67.2
Pearson’s correlation *r*	0.991
Paired *t* test	t(29) = −1.46, *p* = 0.155
Cohen’s d_z	0.27

The human-annotation reference is the mean of the two annotators’ independently derived rotation estimates, each obtained by feeding manually located seam-feature correspondences into the same Horn closed-form alignment used by the automatic pipeline. The negative mean signed difference indicates the automatic pipeline returns marginally lower values than the manual reference.

Agreement with two reference methods built on different physical principles provides cross-method consistency evidence for the rotation pipeline, but should be interpreted as internal consistency among estimation approaches rather than proof of absolute physical rotational accuracy. The gyroscope reference is instrumented, i.e., a genuinely physical inertial measurement, but carries a sensor-modified ball mass and elasticity together with a ±2% calibration band; the human-annotation reference is vision-only and therefore shares part of its error budget with the automatic pipeline while being free of any ball modification. The marginally higher Pearson’s correlation against the human-annotation reference (0.991 versus 0.987 against the gyroscope) is not evidence that human annotation is more accurate: both references occupy overlapping error bands and the difference reflects the smaller sample (*n* = 30 versus 20) and the restriction of the annotation study to post-impact steady-state frames. The residual ≈3% deviation against both references is consistent with, but does not on its own prove, an intrinsic accuracy limit of the pipeline; a fully independent sub-millimeter optical motion-capture benchmark under real-stroke conditions is identified in the limitations of the study as the next-priority validation step.

### Speed-spin coupling across stroke types

The relationship between ball speed and rotational angular velocity reveals the distribution pattern of energy between translational and rotational motion. This study conducted a correlation analysis of ball speed and rotational angular velocity for 178 sample sets. The scatter distributions for different stroke types are shown in Figure 7.

**Figure 7:**
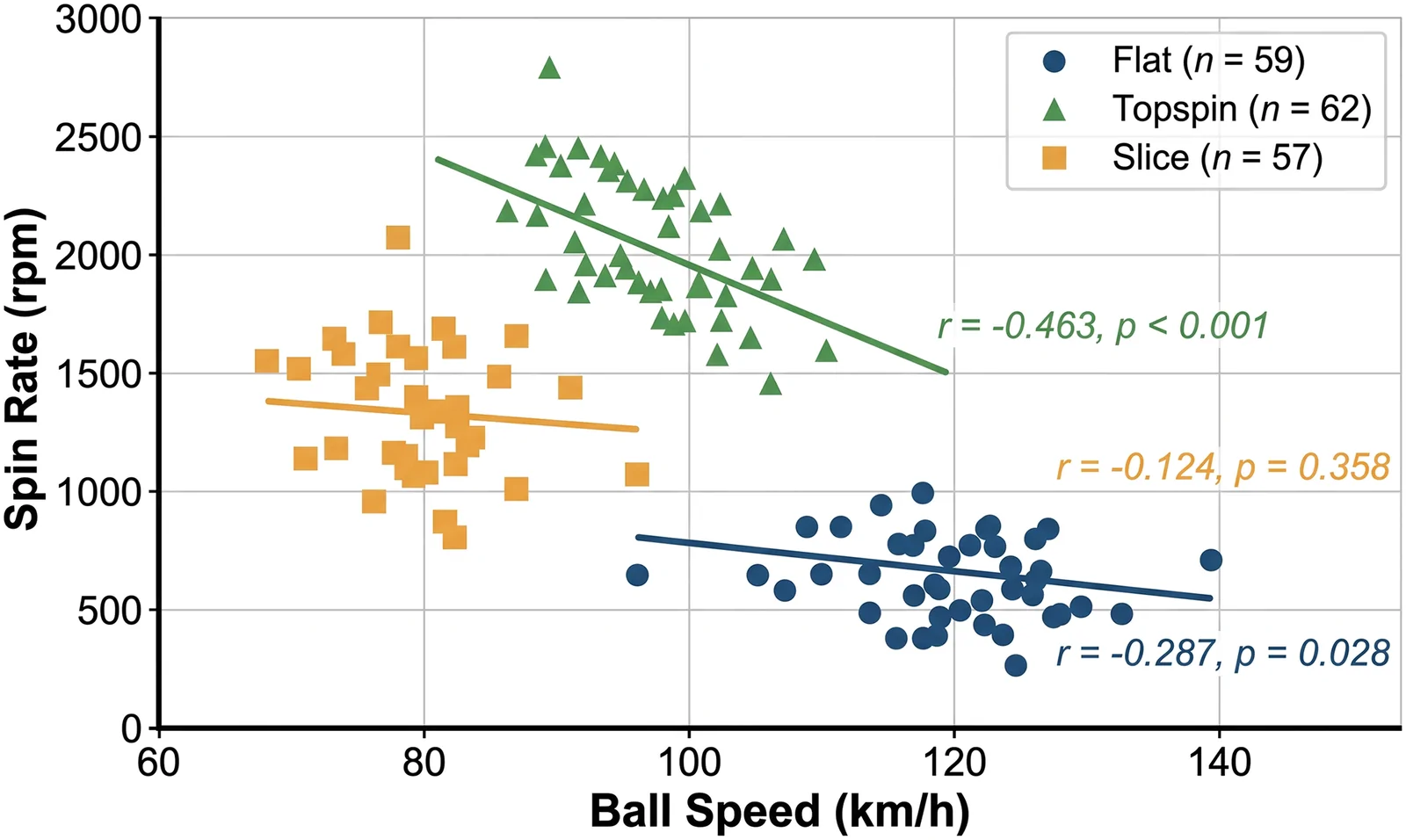
Scatterplot of ball speed versus rotational angular velocity Each point is one valid trial (flat *n* = 59, topspin *n* = 62, slice *n* = 57); lines are per-stroke-type linear fits and r is Pearson’s correlation coefficient with the associated *p* value.

Pearson’s correlation analysis revealed stroke-type-differentiated coupling patterns. Topspin shots showed moderate negative correlation (*r* = −0.463, *p* < 0.001), indicating a speed-spin trade-off where athletes pursuing higher spin typically sacrifice some ball speed. Flat shots showed a weak negative correlation (*r* = −0.287, *p* = 0.028), consistent with the flat technique primarily pursuing speed. No significant correlation was found for slice shots (*r* = −0.124, *p* = 0.358), reflecting tactical flexibility in speed-spin combinations.

To further validate research hypothesis H4, this study constructed a hierarchical regression model with rotational angular velocity as the dependent variable and ball speed, stroke type, and their interaction terms as independent variables. Model comparison results are shown in Table 9.

**Table 9:** Hierarchical regression model comparison of the ball speed-spin coupling relationship

Model	Included variables	R^2^	ΔR^2^	F Change	*p* value
Model 1	ball speed	0.086	0.086	16.53	<0.001
Model 2	ball speed + stroke type	0.724	0.638	198.76	<0.001
Model 3	ball speed + stroke type + interaction terms	0.751	0.027	9.24	<0.001

Dependent variable is rotational angular velocity; *n* = 178. Stroke type is entered as two dummy contrasts with flat shot as reference. ΔR^2^ values are expressed as proportions; equivalently, stroke type accounts for 63.8 percentage points, and the interaction accounts for a further 2.7 percentage points of explained variance.

Model comparison showed that the basic speed-only model (Model 1) had weak explanatory power (*R*^2^ = 0.086), but this should not be read as evidence against speed-spin coupling. It is instead a textbook manifestation of Simpson’s paradox: as Figure 7 shows, the three stroke types occupy distinct vertically offset regions of the speed-spin plane while their within-group regression lines all carry negative slopes, so pooling cancels the within-group trends at the marginal level. The jump from 8.6% (Model 1) to 72.4% (Model 2) once stroke type is admitted as a control means that stroke type as a categorical control absorbed 63.8 percentage points of previously unaccounted variance, and the speed × stroke-type interaction added a further 2.7 percentage points to reach 75.1% (Model 3, *p* < 0.001). The significant interaction term formally decomposes the paradox and confirms differentiated within-group coupling embedded in a heterogeneous pooled sample, supporting H4.

### Test-retest reliability and validity

Reliability assessment of the measurement system employed the test-retest reliability method. The same 15 participants described in the STAR Methods underwent repeated testing one week apart, with each person completing 10 strokes for each stroke type in each session, i.e., approximately 450 strokes per session. After applying the identical YOLO-confidence and stereo-completeness quality control used for the main dataset, the retained trials were aggregated to the participant × stroke-type level, and intraclass correlation coefficients were computed on these paired participant-level means (*n* = 15 pairs per row of Table 10) for both ball speed and rotational angular velocity. Reliability assessment results are shown in Table 10.

**Table 10:** Measurement system test-retest reliability assessment results

Measurement indicator	ICC(2,1)	95% confidence interval	SEM	MDC_95_
Flat shot ball speed	0.924	[0.876, 0.958]	3.42 km/h	9.48 km/h
Topspin shot ball speed	0.908	[0.851, 0.947]	3.28 km/h	9.09 km/h
Slice shot ball speed	0.916	[0.862, 0.952]	2.79 km/h	7.73 km/h
Flat shot rotational angular velocity	0.867	[0.786, 0.921]	68.4 rpm	189.6 rpm
Topspin shot rotational angular velocity	0.891	[0.824, 0.938]	135.8 rpm	376.5 rpm
Slice shot rotational angular velocity	0.883	[0.809, 0.932]	112.3 rpm	311.3 rpm

SEM is the standard error of measurement, and MDC_95_ is the minimal detectable change at 95% confidence, computed as 1.96 × √2 × SEM. ICCs were computed on paired participant × stroke-type means from the two sessions (*n* = 15 pairs per row).

Ball speed ICC values ranged from 0.908 to 0.924, falling within the “excellent” level (ICC >0.90) per the criteria of Koo and Li,^23^ indicating high test-retest stability. Rotational angular velocity ICC ranged from 0.867 to 0.891, falling within “good” (0.75–0.90), slightly lower than ball speed reliability due to spin measurement’s higher sensitivity to image quality and algorithm parameters and to inter-day variation in subjects’ spin control.

### Comparison with existing measurement technologies

To position the present system relative to existing measurement technologies, comparison was made with the commercial Hawk-Eye platform. Since Hawk-Eye raw data are not directly accessible, public ball-speed and spin statistics from professional tournaments served as the comparison reference. System performance positioning is shown in Figure 8; the transparent scoring rubric used to place each platform on the six capability axes is given in the figure legend and in the STAR Methods, so that every axis can be traced back to a measurable quantity rather than a subjective rating.

**Figure 8:**
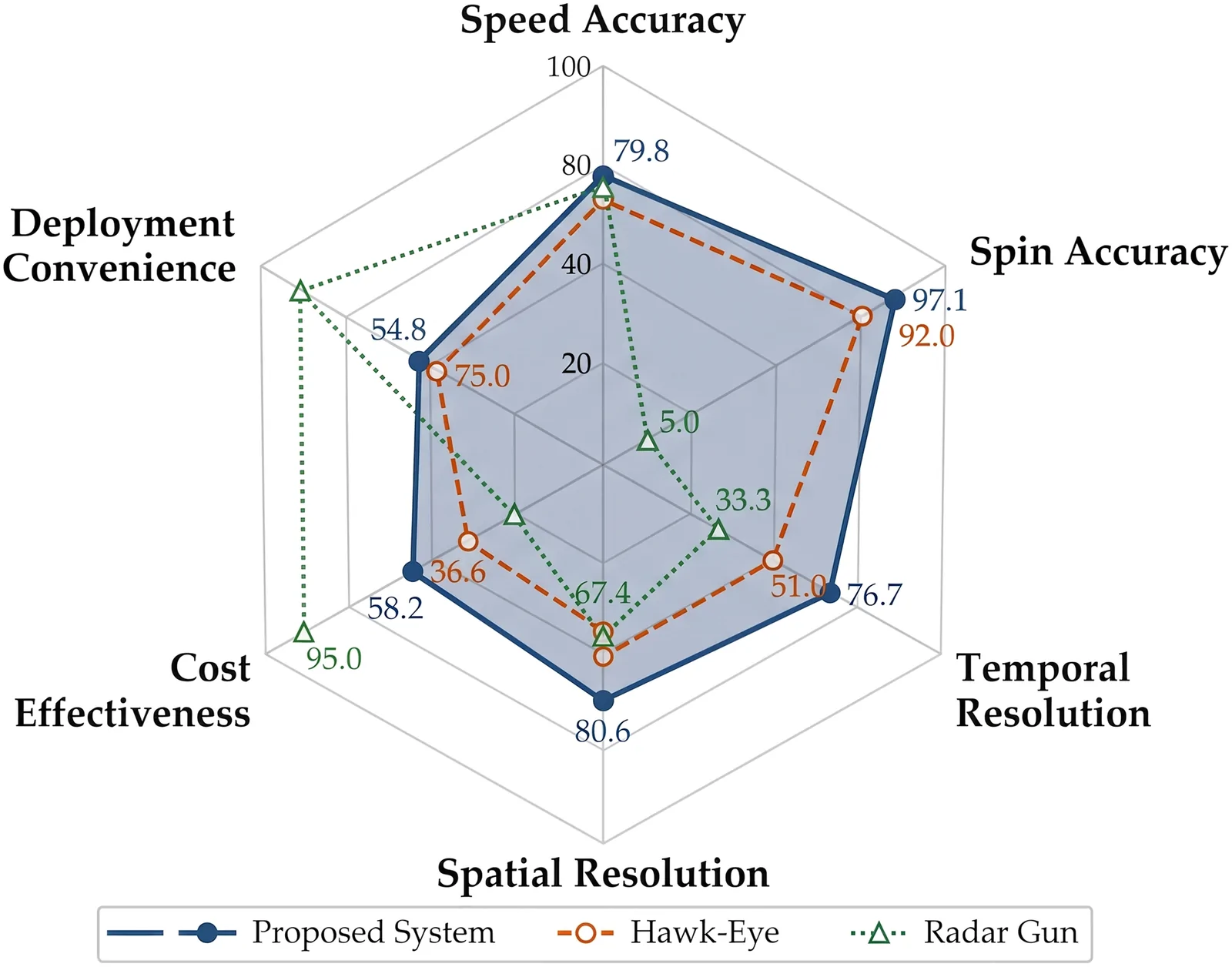
Radar chart comparing this system with existing measurement technologies Six capability axes are each normalized to a 0–10 scale from a measurable quantity, so that no axis rests on a subjective rating. Error-type axes are mapped linearly and inversely: speed accuracy = 10 − speed RMSE in km/h; spatial resolution = 10 × (10 − three-dimensional position RMSE in mm)/9.5 over a 0.5–10 mm reference span; spin accuracy = 10 × (1 − mean absolute deviation rate). Ratio-type axes are mapped logarithmically: temporal resolution = 10 × log_10_(frame rate/10)/log_10_(1000) over a 10–10,000 fps span; deployment convenience = 10 × log_10_(100/operator-hours)/log_10_(100) over a 1–100 operator-hour span, where operator-hours is set-up time multiplied by the number of operators required; and cost-effectiveness = 10 × log_10_(10^6^/acquisition cost in USD)/log_10_(1000) over a USD 10^3^–10^6^ span. Applying these definitions to the present system (Tables 2, 3, 7, 11, and 12) gives 7.98 for speed accuracy, 8.06 for spatial resolution, 9.71 for spin accuracy, 7.67 for temporal resolution, 5.48 for deployment convenience and 5.82 for cost-effectiveness. Values for the commercial platform and the stand-alone radar are computed by the same definitions from published specifications and tournament statistics, so the comparison indicates relative positioning rather than a synchronized same-court benchmark.

From the multi-dimensional capability profile shown in Figure 8, the present system, the commercial Hawk-Eye platform, and the stand-alone radar speed gun occupy different positions in the speed-accuracy/spin-accuracy/temporal-resolution/spatial-resolution/deployment-convenience/cost-effectiveness capability space, reflecting their distinct hardware architectures and intended scenarios. The present system is positioned mainly on the deployment-convenience and cost-effectiveness axes, with the hardware bill of materials of approximately RMB 128,000 (Table 11) and on-site assembly by two technicians within 4 h on a single 2 × 2 m hitting zone. Its capability profile is not directly substitutable for a full-court Hawk-Eye installation, and a direct, point-for-point quantitative performance comparison would require a synchronized same-court raw-data validation campaign, which lies outside the data-access licensing available for the present study. The platforms also differ structurally in field-of-view coverage, simultaneously tracked events, and end-user workflow: a Hawk-Eye installation deploys six to eight cameras around the full court for trajectory tracking and line-call adjudication, while the present binocular configuration is confined to a single hitting zone for fine-grained ball-speed and spin measurement around the impact phase. The proper framing is therefore functional complementarity rather than substitution. The present system is intended for in-court technical diagnosis at one hitting zone in research, education, and grassroots-training settings, while full-court trajectory tracking and elite-tournament line-call adjudication remain the proper domain of multi-camera commercial platforms. This positioning makes the present configuration suitable for grassroots training units and teaching institutions within its single-hitting-zone scope, providing a cost-effective in-court tennis technical analysis solution.

**Table 11:** Itemized hardware cost composition of the proposed system

Equipment category	Specification	Unit price (RMB)	Quantity	Subtotal (RMB)
Industrial high-speed camera	1-inch CMOS, 2000 fps, global shutter	28,000–35,000	2	≈63,000
Fixed-focal-length lens	35 mm, f/1.4	6,000–8,000	2	≈14,000
FPGA synchronous trigger	Xilinx Spartan-6 pulse generator	8,000	1	8,000
LED constant-current illumination	5600 K, 95% uniformity	5,000	4	20,000
Data-acquisition workstation	Intel i7-12700K + NVIDIA RTX 3060	15,000	1	15,000
Infrared light-curtain sensor	trigger pulse generator	3,000	1	3,000
Calibration target and cabling	checkerboard target, camera link cables	5,000	1	5,000
Total	–	–	–	≈128,000

Prices are 2025–2026 Chinese market list prices for industrial machine-vision components; subtotals for the camera and lens use the midpoint of the quoted unit-price range. The total of ≈RMB 128,000 corresponds to ≈ USD 18,000 at an assumed rate of 1 USD ≈7.1 RMB.

### Runtime and computational complexity

The pipeline ingests stereo streams at 2000 fps per camera and is intended for in-training feedback rather than purely offline post-analysis, so the per-frame computational cost was profiled on a single workstation (Intel Core i7-12700K, NVIDIA RTX 3060 12 GB; 1280 × 800 px 8-bit grayscale). The per-frame wall-clock cost, averaged over 5000 frames from the YOLO validation set (excluding disk I/O), is summarized in Table 12. Preprocessing, deblurring, detection, stereo matching, feature detection, and Lucas-Kanade tracking were executed on the GPU (CUDA 12, TensorRT FP16); three-dimensional reconstruction, Kalman filtering, and quaternion update were retained on the CPU since they operate on low-dimensional problems for which CPU latency is lower than GPU kernel-launch overhead.

**Table 12:** Per-frame runtime and computational complexity of the image-processing pipeline

Processing module	Hardware	Per-frame time (ms)	Asymptotic complexity
Image preprocessing (denoising, distortion correction)	GPU (CUDA)	2.1	O(WH)
Motion-blur deblurring (Richardson-Lucy on ROI)	GPU (CUDA)	1.4	O(WᵣHᵣ)
YOLOv8-s ball detection	GPU (TensorRT FP16)	4.8	O(C·WH)
Stereo matching and disparity computation	GPU (CUDA)	1.2	O(WH·d)
Three-dimensional coordinate reconstruction	CPU	0.3	O(1)
Trajectory fitting and Kalman filtering	CPU	0.5	O(1)
Shi-Tomasi surface-feature detection	GPU (CUDA)	3.6	O(WH)
Pyramidal Lucas-Kanade tracking	GPU (CUDA)	2.8	O(K·win^2^·L)
Horn closed-form attitude estimation	CPU	1.1	O(K^3^)
Total per-frame processing time	–	≈17.8	–

Symbols: *W* = image width (1280 px); H = image height (800 px); Wᵣ, Hᵣ = ROI width and height; *C* = detector channel count; *d* = disparity search range; *K* = number of tracked surface feature points; win = Lucas-Kanade integration-window side; *L* = pyramid level count. Aggregate 17.8 ms per frame corresponds to a sustained ≈56 fps on the profiling workstation. GPU-resident stages account for 15.9 ms and CPU-resident stages for 1.9 ms of the total.

At an aggregate cost of approximately 17.8 ms per frame, the pipeline reaches a sustained processing rate of roughly 56 fps, sufficient for real-time playback at conventional video rates but falling short of the 2000 fps acquisition rate by a factor of ≈36. It should be emphasized that the 2000 fps acquisition rate is dictated by the underlying physics rather than by any real-time output requirement: the 4–5 ms racket-ball contact demands ≥1000 fps sampling for single-frame impact localization and sub-diameter inter-frame ball displacement. The system therefore operates in a post-stroke batch mode. Each captured stroke is downloaded from the camera buffer to the NVMe SSD array and processed on a bounded frame budget, returning the consolidated speed-and-spin readout to the coach interface approximately 3 s after contact. This latency decomposes as ≈0.6 s for burst download of the two 201-frame bursts over Camera Link Full, ≈1.0 s for the coarse detection pass that localizes the impact frame across the acquisition window, and ≈1.1 s for the full pipeline on the ≈60 frames that actually carry the speed and rotation measurement. Three seconds is comfortably inside the 25 s between-point interval permitted in tennis, so the readout is available to the coach before the next rally begins. The software layer in Figure 9 is accordingly labeled “Image Acquisition and Buffering Software”: the real-time component performs hardware-synchronized acquisition, distortion correction, and high-throughput buffering, while the heavier per-frame detection, stereo-reconstruction, and rotation-estimation are executed on the buffered sequence.

**Figure 9:**
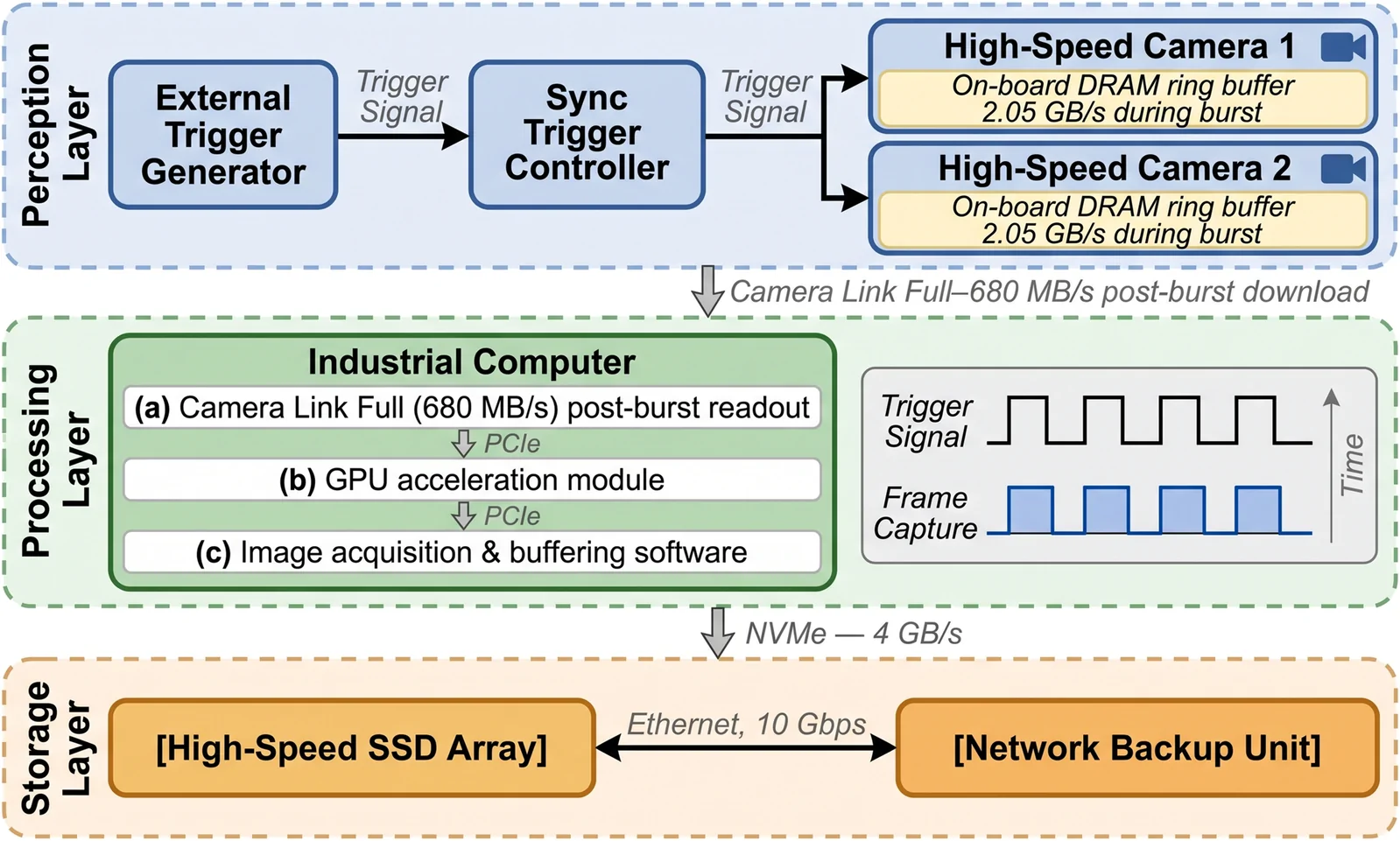
Ultra-high-speed photography system hardware architecture Hardware architecture of the binocular ultra-high-speed photography system, including the two 1-inch-CMOS 2000 fps industrial cameras with matched 35 mm f/1.4 lenses, the Xilinx Spartan-6 FPGA synchronous trigger, the LED illumination array, the infrared light-curtain and the data-acquisition workstation.

Three avenues are available for closing the gap toward true frame-rate processing if hard real-time feedback during extended rallies becomes required, and their contributions are additive in a transparent way. First, hardware: migrating to a recent data-center GPU such as NVIDIA L4 or RTX 6000 Ada roughly quadruples detector and dense-optical-flow throughput, which reduces the 15.9 ms GPU-resident portion of Table 12 to ≈4.0 ms and lifts the pipeline from 56 fps to ≈170 fps. Second, dedicated logic: implementing the Shi-Tomasi and Lucas-Kanade kernels on an FPGA co-processor removes both their residual cost and the PCIe transfer overhead, giving ≈230 fps. Third, algorithmic: event-triggered processing that fully analyses only the ±35-frame window bracketing each detected impact—the 27-frame deformation-exclusion core plus the 20-frame steady-state rotation window on either side—and samples the intervening free-flight phase at one frame in five yields an asymptotic ≈5× gain in effective throughput, for an equivalent ≈1200 fps. The ≥1000 fps sampling criterion is not violated by this last step because it applies to the impact bracket, where the velocity vector changes discontinuously; during smooth ballistic flight the Kalman predictor bridges the sparser samples. Together, these three routes address the great majority of the ≈36× gap; closing the final factor of roughly two would require either multi-GPU parallelism or a full FPGA/ASIC realization of the detection stage, and is left to a later iteration.

## Discussion

The ultra-high-speed photography measurement system achieved the expected synchronized measurement of ball speed and spin parameters around the impact phase. Experimental data systematically validated the four research hypotheses, with results summarized in Table 13.The system was operated under the technical settings listed in Table 14 and across the three instructed stroke conditions listed in Table 15. Data were collected in the indoor court configuration of Figure 10, and all reported speed and spin values were produced by the image-processing and parameter-extraction pipeline of Figure 11 together with the seam-texture rotation principle of Figure 12.

**Table 13:** Summary of research hypothesis test results

Hypothesis	Hypothesis content	Key validation indicator	Actual result	Threshold	Test conclusion
H1	ball-center three-dimensional reconstruction accuracy ≤3 mm around the impact phase	static checkerboard and dynamic linear-stage 3-D position RMSE against independent references	static 1.83 mm; dynamic pooled 2.34 mm	≤3 mm	supported
H2	spin measurement accuracy ≤ ±5% during the post-contact steady-state flight window	angular-velocity mean absolute deviation rate against the smart-ball gyroscope and the blinded human-annotation reference	2.87% (gyroscope); 3.05% (human annotation)	≤5%	supported (within the pre-defined ±5% margin as cross-method consistency; absolute rotational accuracy awaits sub-millimeter optical motion-capture benchmarking)
H3	reference-system agreement within the pre-defined practically acceptable margin	Bland-Altman mean bias with 95% CI and equivalence margin; supporting paired *t* test with effect size	ball speed: bias 0.44 km/h, 95% CI [−0.02, +0.90], t(177) = 1.87, *p* = 0.063, d_z = 0.14; spin: t(19) = 1.89, *p* = 0.074	Bias and its 95% CI within ±2.5 km/h; *p* > 0.05	supported (no clear evidence of practically meaningful bias within the tested range and within the pre-defined equivalence margin)
H4	ball speed-spin coupling relationship exists during the post-contact steady-state flight window	interaction term *F* test	*F* change = 9.24, ΔR^2^ = 0.027, *p* < 0.001	*p* < 0.05	supported

**Table 14:** Main technical parameters of the ultra-high-speed photography system

Parameter category	Parameter name	Parameter value	Remarks
Image sensor	sensor type	CMOS	global shutter
Image sensor	pixel size	10 μm	–
Image sensor	resolution	1280 × 800 pixels	at 2000 fps
Optical system	lens focal length	35 mm	fixed focal lens
Optical system	maximum aperture	f/1.4	–
Temporal characteristics	maximum frame rate	5000 fps	reduced-resolution mode
Temporal characteristics	exposure time	100 μs	adjustable range 1–500 μs
Synchronization performance	trigger delay	<1 μs (measured 0.81 ± 0.04 μs)	external trigger mode
Synchronization performance	inter-camera frame-onset offset	<100 ns (measured 67 ± 32 ns)	across 200 LED-pulse events
Data path	full-rate acquisition	2.05 GB/s per camera	written to on-board DRAM ring buffer
Data path	download interface	camera link Full, 680 MB/s	post-burst download of the buffered ±50 ms window (201 frames ≈206 MB per camera)

Sustained 1280 × 800 px 8-bit acquisition at 2000 fps generates 2.05 GB/s per camera, which exceeds the Camera Link Full link rate; the architecture therefore buffers each triggered burst to on-board camera DRAM at full rate and downloads it afterward, which is the reason the pipeline operates in post-stroke batch mode.

**Table 15:** Experimental design condition summary

Condition number	Stroke type	Nominal instructed ball-speed range	Expected spin characteristics	Retained trials
C1	flat shot	100–130 km/h	low spin	≈4 per participant (59 retained in total)
C2	topspin shot	80–110 km/h	forward high spin	≈4 per participant (62 retained in total)
C3	slice shot	70–100 km/h	reverse medium spin	≈4 per participant (57 retained in total)

The ball-speed ranges are the nominal targets communicated to participants as task instructions; the ball speeds actually achieved are reported in Table 1 and extend somewhat beyond these ranges in both directions, as expected for freely executed strokes. Retained-trial counts are the post-quality-control totals analyzed in the results (178 trials in total).

**Figure 10:**
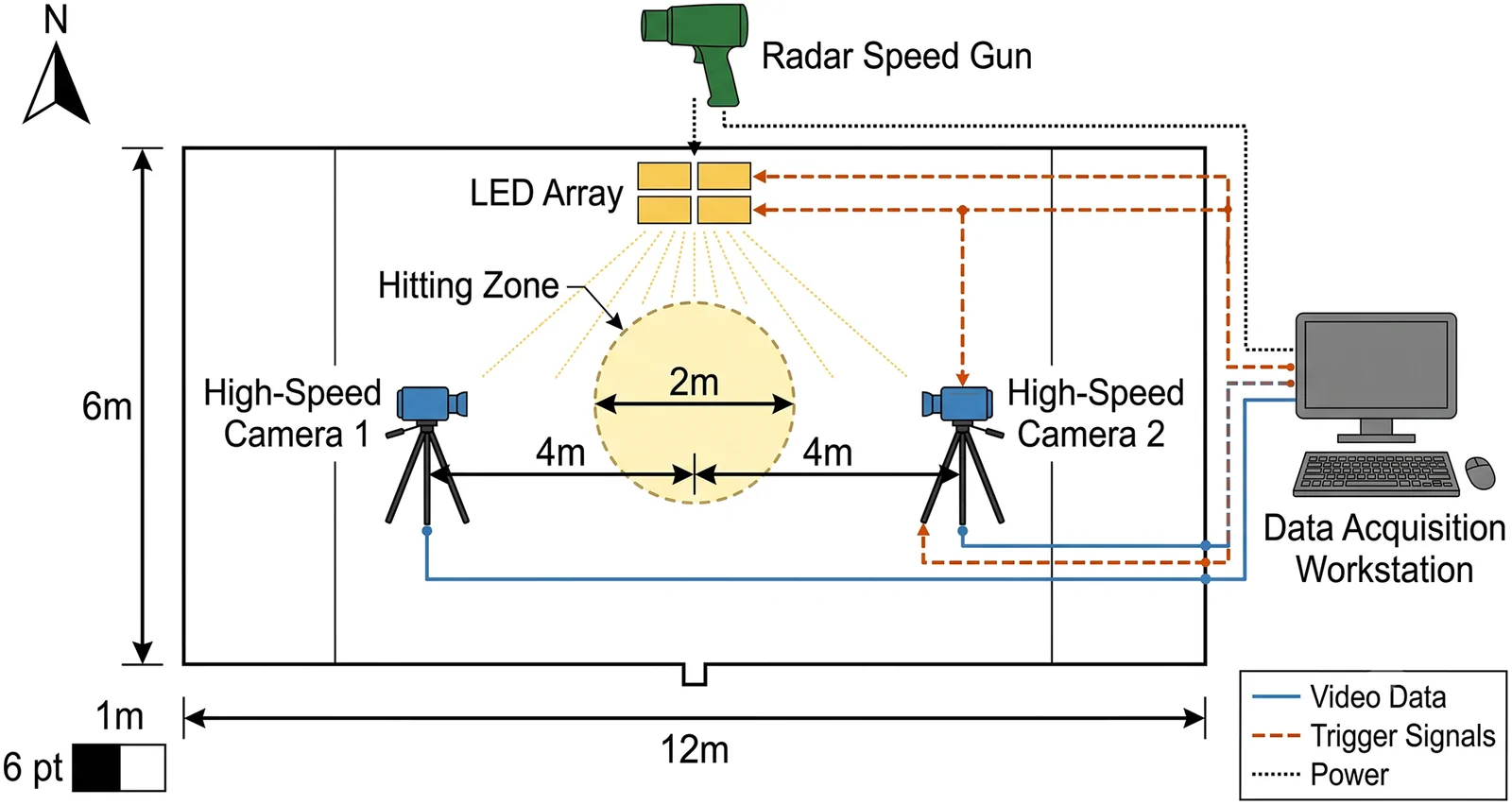
Experimental venue layout and equipment configuration Layout of the indoor standard tennis court, the binocular camera positions and the four LED constant-current illumination arrays used for data collection.

**Figure 11:**
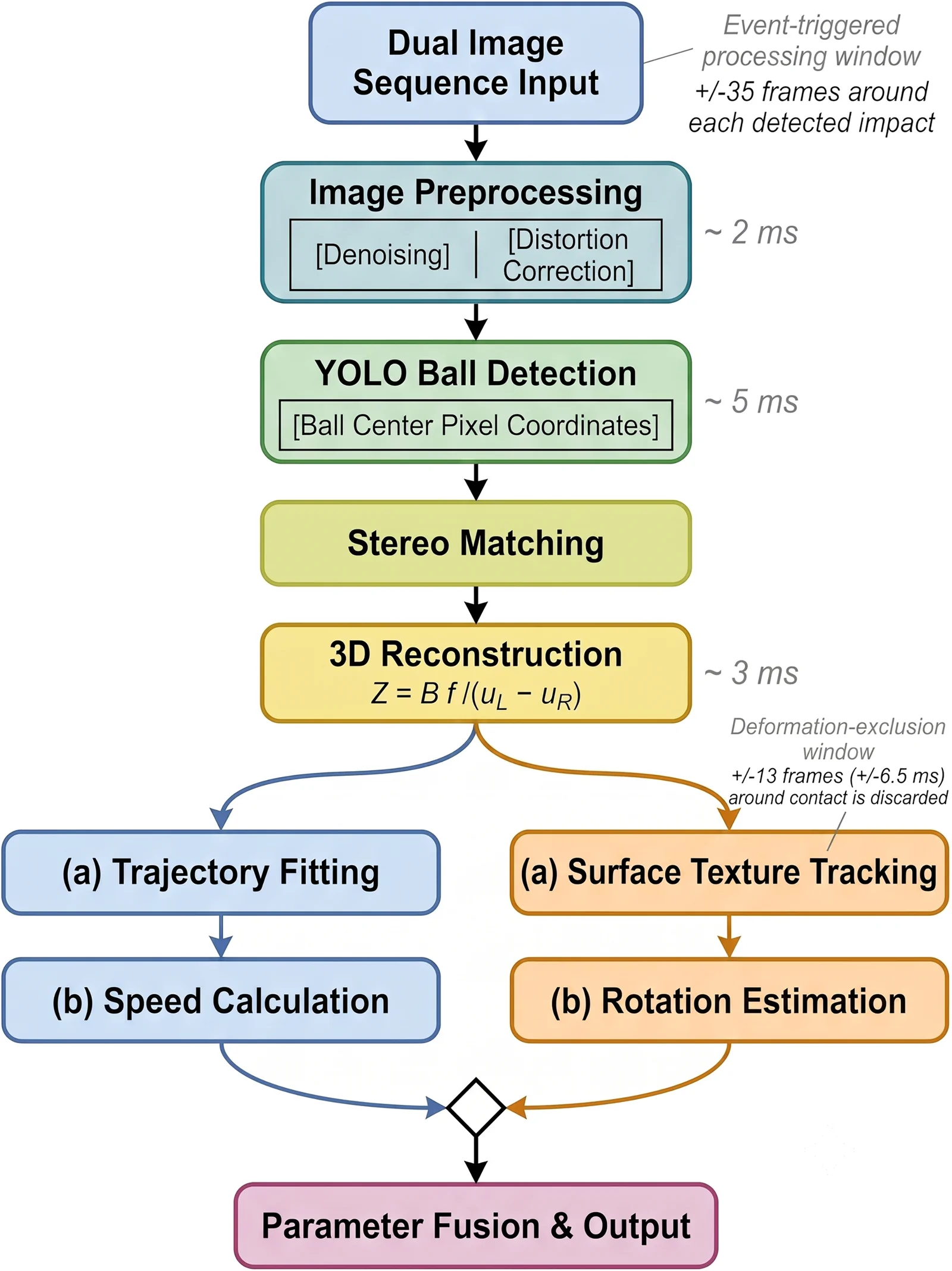
Image processing and parameter extraction algorithm flowchart Flowchart of the four-module image-processing and parameter-extraction pipeline: ball detection, three-dimensional reconstruction, speed calculation, and spin estimation.

**Figure 12:**
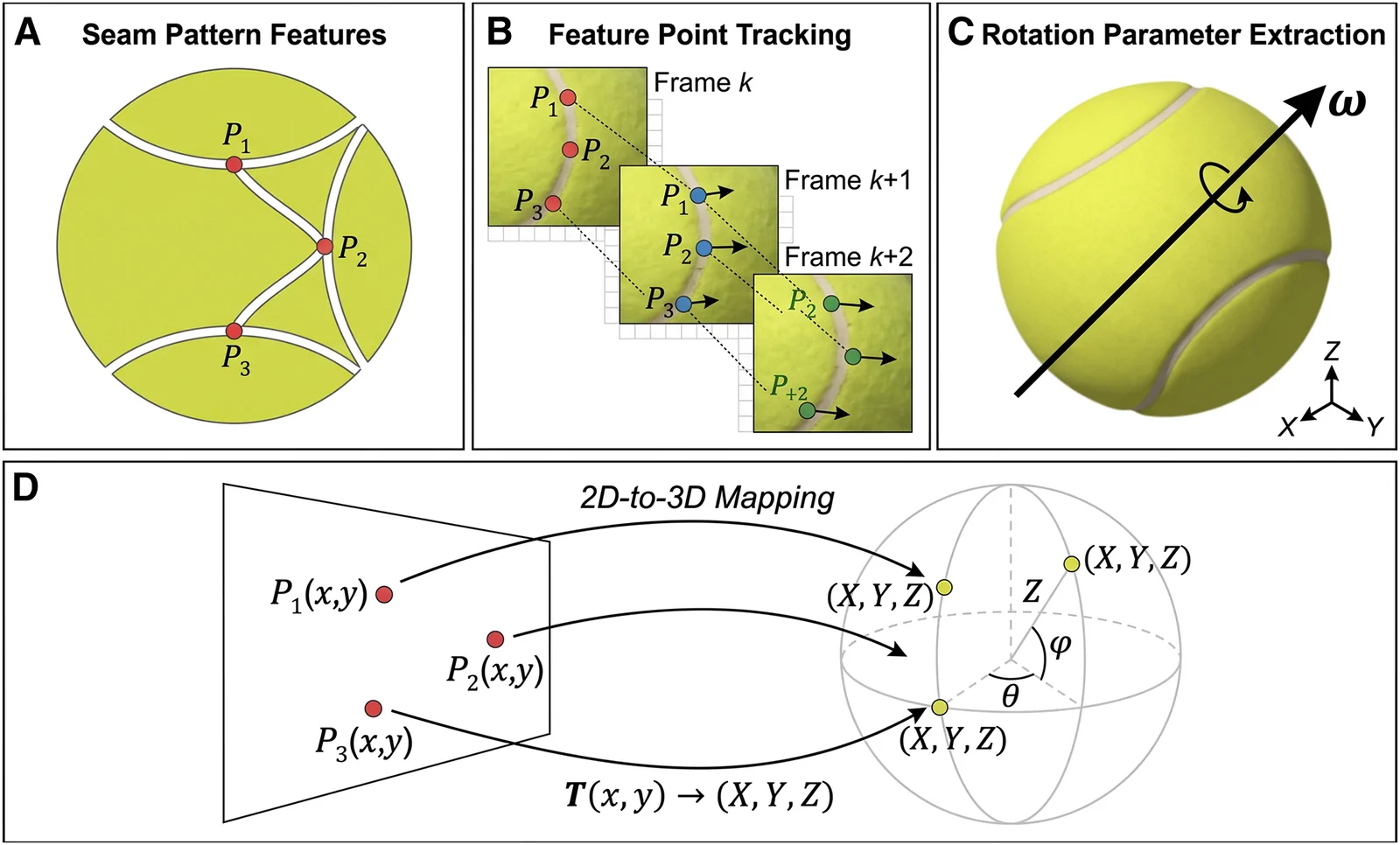
Ball surface texture tracking principle Schematic of the ball-surface-texture-tracking principle used to recover rotation from the natural seam pattern of a standard ITF tennis ball.

For hypothesis H1, the absolute accuracy of three-dimensional ball-center reconstruction was verified through the independent static and dynamic calibration experiments described in the STAR Methods and the results, rather than through indirect back-calculation from ball-speed RMSE. The static checkerboard experiment yielded a combined three-dimensional position RMSE of 1.83 mm; the dynamic linear-stage experiment yielded a pooled three-dimensional position RMSE of 2.34 mm and a speed RMSE of 0.40 km/h across the 5–20 m/s range. Both values lie below the 3 mm H1 threshold and are not derived from any quantity produced by the downstream ball-speed pipeline, removing the circular-logic concern raised at review.

Hypothesis H2 was supported within its pre-defined ±5% threshold, with a rotational angular velocity mean absolute deviation rate of 2.87% against the smart-ball gyroscope and spin-type recognition accuracy of 93.3%. This extends existing spin measurement: previous research mostly relied on artificial markers or custom balls, while this study shows reliable spin information can be obtained through high-resolution image analysis of natural seam texture. The smart tennis ball remains an instrumented sensor with a ±2% gyroscope-level error band and fixed-point ball-machine constraint. A sensor-independent blinded human-annotation reference (30 trials, ICC(2,k) = 0.984) produced a mean absolute deviation rate of 3.05%, Pearson’s *r* = 0.991, and a non-significant paired *t*-test (t(29) = −1.46, *p* = 0.155), providing cross-method consistency evidence rather than proof of absolute rotational accuracy. To our knowledge, this is the first tennis-spin study to report agreement against two reference systems built on fundamentally different physical principles—an inertial measurement and a vision-only human annotation—on the same set of hitting trials. Sub-millimeter optical motion-capture under real-stroke conditions is identified as the highest-priority follow-up validation.

Validation results for H3 are interpreted within the Bland-Altman framework of the STAR Methods. The mean camera-radar bias was 0.44 km/h with a cluster-corrected 95% CI of the bias of [−0.02, +0.90] km/h, both lying within the pre-defined practically acceptable equivalence margin of ±2.5 km/h; the 95% limits of agreement [−3.44, +4.32] km/h, computed from the raw paired-difference standard deviation as Bland-Altman practice requires, are slightly wider than this margin (by 0.94 and 1.82 km/h), reflecting natural inter-trial stroke variability rather than systematic instrument offset. The supporting cluster-corrected paired *t* test (t(177) = 1.87, *p* = 0.063, Cohen’s d_z = 0.14) and Bayesian indicator BF01 = 2.81 are mutually consistent. The data therefore provide no clear evidence of practically meaningful bias within the tested 63.8–142.7 km/h range; the conclusion is framed in terms of the ±2.5 km/h margin rather than the radar’s ±0.16 km/h nominal accuracy, since the upper bound of the bias 95% CI (+0.90 km/h) exceeds the radar accuracy band. The smart-ball-gyroscope comparison (t(19) = 1.89, *p* = 0.074) supports the same conclusion for rotational angular velocity with the same consistency-versus-absolute-accuracy caveat as for H2.

H4 was validated through hierarchical regression. The complete model containing ball speed, stroke type, and their interaction explains 75.1% of rotational angular velocity variance; the interaction is significant (ΔR^2^ = 0.027, F change = 9.24, *p* < 0.001). Topspin shows moderate negative correlation (*r* = −0.463), flat weak negative (*r* = −0.287), and slice shows no significant correlation (*r* = −0.124). The weak speed-only Model 1 (*R*^2^ = 0.086) does not contradict this: as analyzed under speed-spin coupling across stroke types, the pooled marginal association is suppressed by Simpson’s paradox, because the three stroke groups occupy distinct vertically offset regions of the speed-spin plane while sharing within-group negative slopes. The jump from 8.6% to 75.1% once stroke type and its interaction with speed are admitted is the signature of differentiated within-group coupling rather than absent coupling, providing preliminary quantitative reference for coaches to formulate differentiated training strategies.

This study addressed the precise measurement of ball speed and rotational angular velocity around the impact phase in tennis through a synchronized binocular ultra-high-speed photography system, validated across flat, topspin, and slice strokes. The main conclusions are as follows. First, the system captures impact details at 2000 frames/second, with three-dimensional reconstruction accuracy independently verified through static checkerboard calibration (1.83 mm position RMSE) and dynamic linear-stage calibration (2.34 mm pooled RMSE across 5–20 m/s); ball-speed RMSE is 2.02 km/h with MAPE 1.97%, with a Bland-Altman mean camera-radar bias of 0.44 km/h and cluster-corrected 95% CI of the bias [−0.02, +0.90] km/h, both lying within the pre-defined ±2.5 km/h equivalence margin, providing no clear evidence of practically meaningful bias within the tested 63.8–142.7 km/h range (supporting indicators: *p* = 0.063, Cohen’s d_z = 0.14, BF01 = 2.81). Second, the surface-texture-tracking spin estimator achieved non-contact measurement on standard ITF balls with mean absolute deviation rates of 2.87% (gyroscope) and 3.05% (human annotation, ICC(2,k) = 0.984), and spin-type recognition accuracy 93.3%; agreement against the two references provides cross-method consistency rather than absolute physical rotational accuracy. Third, differentiated speed-spin coupling exists across stroke types during the post-contact steady-state flight window (topspin *r* = −0.463, flat *r* = −0.287, slice *r* = −0.124), with the regression model containing interaction terms explaining 75.1% of rotational angular velocity variance, providing a measurement-side foundation and preliminary quantitative reference for technique evaluation and differentiated training design rather than a finalized theoretical basis, since causal training-effect claims await longitudinal intervention studies.

Three methodological contributions warrant explicit summary. The independent three-dimensional reconstruction validation removes the earlier circular reasoning between reconstruction accuracy and ball-speed RMSE and supplies direct mechanism-level evidence for the spatial-accuracy hypothesis. The cross-validation of the rotation estimator against two reference systems built on different physical principles documents the cross-method consistency of the rotation-estimation pipeline at the ≈3% deviation level, while leaving the absolute rotational accuracy benchmark to a future sub-millimeter optical motion-capture campaign. The full pipeline operates at approximately 17.8 ms per frame on a single workstation-class GPU and acquires its data in a hardware-synchronized manner with 67 ± 32 ns inter-camera frame-onset offset, providing a frame-based alternative to event-camera approaches that retains compatibility with mature computer-vision toolchains.

### Implications

From the training perspective, the speed-spin coupling relationships revealed here provide an analytical framework for technical diagnosis: coaches can identify whether athletes’ technical characteristics conform to typical stroke-type patterns by monitoring speed-spin scatter distributions. Topspin data clustering excessively in the high-speed-low-spin region may indicate insufficient racket face angle or incomplete brushing motion. The distributions of Table 6 and Figure 7 make this operational: a nominally topspin trial returning below ≈1200 rpm while exceeding ≈110 km/h lies more than 1.5 SD outside the topspin cluster on both axes and can be flagged automatically in a coach dashboard for shot-by-shot review, most often reflecting premature racket-face closure or an insufficiently steep brushing path. Multiple regression confirms swing speed as the primary determinant (*β* = 0.612), while racket face vertical angle correlates negatively (β = −0.203), reminding coaches to balance spin generation against speed retention.

From the technology perspective, this study validated the feasibility of medium-cost ultra-high-speed photography in professional sports measurement. The hardware bill of materials (Table 11) totals approximately RMB 128,000 (≈USD 18,000), and the system can be deployed by two technicians within 4 h. A direct cost ratio against a commercial Hawk-Eye installation is avoided as misleading: Hawk-Eye typically deploys six to eight cameras around the full court for trajectory tracking and line-call adjudication, whereas the present binocular configuration is confined to a single 2 × 2 m hitting zone for fine-grained kinematic measurement around the impact phase. The framing is therefore functional complementarity rather than substitution: the present system targets in-court technical diagnosis at one hitting zone in research, education, and grassroots settings, while full-court trajectory tracking and elite-tournament adjudication remain the domain of multi-camera commercial platforms. Elite first serves above 200 km/h sit at the upper bound of the configuration and would require a sensor upgrade.

On the scientific side, the engineering-application value should not be conflated with biomechanical novelty. The rank ordering of speed and rotation across flat, topspin and slice strokes is consistent with long-standing tennis-biomechanics observations and serves as cross-check evidence. The primary contributions are methodological: (1) quantifying within-stroke-type speed-spin coupling coefficients on the same hitting trials through a single synchronized pipeline (topspin *r* = −0.463, flat *r* = −0.287, slice *r* = −0.124), providing organism-level reference values that literature relying on separate devices does not directly furnish; (2) showing that a frame-based 2000 fps binocular configuration with Horn-style closed-form attitude estimation can deliver ±5%-class rotation accuracy on the natural seam pattern of a standard ITF ball without markers, custom balls or event-camera hardware; and (3) merging speed and spin extraction into a single visual pipeline, removing inter-device synchronization error.

### Limitations of the study

This study has several limitations. The 15-participant cohort (12 male and 3 female, mean age 22.3 ± 3.1 years, all right-handed) is dominated by male and right-handed players, so sex-balanced and laterality-balanced extension is needed before the present distributions can be generalized to broader populations. Because the 178 trials are nested within these 15 participants, trial-level ordinary least-squares estimates were cross-checked against linear mixed-effects models (participant-level ICC = 0.18 for ball speed and 0.16 for rotational angular velocity, all fixed-effect deviations below the 5% flag threshold); nonetheless, a larger cohort is required before player-level random effects can be modeled with full confidence. The validation framework also carries irreducible boundary conditions: the smart-ball gyroscope has a ±2% calibration band and modified ball mechanics, and the blinded human-annotation reference is itself vision-based and shares part of its error budget with the automatic pipeline, so both references provide cross-method consistency rather than absolute ground truth. Sub-millimeter optical motion-capture under real-stroke conditions is therefore identified as the next-priority benchmark.

A further set of scope conditions bounds the present claims. The rotation-estimation conclusion is restricted to the steady-state flight window excluding the ±13-frame ball-deformation interval; the strict contact-instant rotation, which would require coupled deformation-rotation modeling, is not claimed. All measurements were obtained on new, clean, yellow ITF-certified competition balls under controlled indoor illumination; ball color (yellow versus orange training balls) and wear stage modify seam-pattern contrast and could in principle shift the Shi-Tomasi feature-point yield, so cross-ball-type and wear-stage generalizability warrants a dedicated ablation. The configuration is dedicated to localized single-zone measurement (2 × 2 m) rather than whole-court trajectory tracking, scoping it to research, education, and grassroots-training diagnosis. Finally, the system currently performs post-stroke batch processing with an ≈3 s coach-facing latency rather than true frame-rate real-time feedback, although a hardware, dedicated-logic, and algorithmic route toward frame-rate operation is quantified in the runtime and computational complexity analysis. Addressing these points (expanding cohort coverage, adding optical motion-capture cross-validation, running a ball-condition ablation, and developing an embedded real-time module) constitutes the principal direction of future work.

## Resource availability

### Lead contact

Further information and requests for resources and reagents should be directed to and will be fulfilled by the Lead Contact, Bin Lv (344587278@163.com).

### Materials availability

This study did not generate new unique reagents. The measurement system was assembled from the commercially available and custom components listed in the key resources table.

### Data and code availability

• The ball-speed and rotational-angular-velocity datasets reported in this paper will be shared by the lead contact upon request; any accession numbers, once the data are deposited in a public repository, will be listed in the key resources table.
• The purpose-built tennis-ball detection dataset (6,500 annotated images) and the fine-tuned YOLOv8-s detector weights used in this study will be shared by the lead contact upon request.
• All original image-processing, three-dimensional-reconstruction and rotation-estimation code will be shared by the lead contact upon request; any accession numbers, once the code is deposited in a public repository, will be listed in the key resources table.
• Any additional information required to reanalyze the data reported in this paper is available from the lead contact upon request.

## Acknowledgments

This research received no external funding. The authors thank the 15 tennis players from the Beijing provincial tennis training center and the varsity team of Capital University of Physical Education and Sports for their participation, and the laboratory technical staff for assistance with camera calibration and the construction of the canonical seam template.

## Author contributions

B.S.: writing – original draft, methodology, data curation, and conceptualization. B.L.: writing – review and editing, validation, supervision, and conceptualization. Y.W.: conceptualization, supervision, validation, and investigation.

## Declaration of interests

The authors declare no competing interests.

## STAR★Methods

### Key resources table

**Table:** 

REAGENT or RESOURCE	SOURCE	IDENTIFIER
**Deposited data**		

Ball-speed and rotational-angular-velocity dataset (178 valid strokes)	This study	N/A (available from the lead contact upon request)
Tennis-ball detection dataset (6,500 annotated images) and fine-tuned YOLOv8-s weights	This study	N/A (available from the lead contact upon request)

**Software and algorithms**		

YOLOv8-s object detector, fine-tuned for tennis-ball detection	Ultralytics; this study	https://github.com/ultralytics/ultralytics
OpenCV (Shi–Tomasi detector, pyramidal Lucas–Kanade tracker)	OpenCV	https://opencv.org
IBM SPSS Statistics 26.0	IBM	https://www.ibm.com/spss; RRID: SCR_002865
R 4.3.2 with the lme4 package	R Core Team; Bates et al.^24^	https://www.r-project.org; CRAN: lme4; RRID: SCR_001905; RRID: SCR_015654
LabelImg image-annotation tool	Open source (Tzutalin)	https://github.com/HumanSignal/labelImg
Original image-processing, 3-D reconstruction and rotation-estimation pipeline	This study	N/A (available from the lead contact upon request)

**Other**		

Industrial high-speed camera, 1-inch CMOS global shutter, 1280 × 800 @ 2000 fps, 10 μm pixel, on-board DRAM ring buffer, Camera Link Full download	Revealer (Hefei Fuhuang AgileDevice)	Revealer X113M (1-inch CMOS global shutter, 1280 × 800 @ 2000 fps, Camera Link Full, on-board DRAM ring buffer); Hefei Fuhuang AgileDevice Co., Ltd., China
Fixed-focal-length lens, 35 mm, f/1.4, ≥1-inch image circle, C-mount	Computar (CBC Group)	Computar V3514-MP (35 mm, f/1.4, 1-inch image circle, C-mount); CBC Group
LED constant-current illumination array, 5600 K, >95% uniformity (4 panels)	Machine-vision LED vendor	5600 K constant-current LED panel; 4-unit array
Smart tennis ball with embedded gyroscope (nominal ±2%)	Custom-built, gyroscope-instrumented (cf. Foo et al., 2024)^9^	N/A (research-grade; fixed-point throwing only)
Synchronous trigger FPGA	AMD/Xilinx	XC6SLX9 (Spartan-6)
Radar speed gun	Applied Concepts (Stalker)	Stalker ATS II
ITF-certified competition tennis balls, 67 mm, 57 g (new, yellow)	Wilson Sporting Goods Co.	Wilson US Open Extra Duty (ITF-approved, Type 2, yellow, 67 mm, 57 g); Wilson Sporting Goods Co.
Precision servo-driven linear stage (0.1 μm encoder, ±20 μm repeatability)	Aerotech Inc.	Aerotech PRO165LM linear-motor stage with Heidenhain LC 495 optical encoder (0.1 μm resolution, ±20 μm bidirectional repeatability); Aerotech Inc.
Precision three-axis checkerboard calibration target (24 corners certified ±0.05 mm)	Applied Image Inc.	Applied Image T-20A precision checkerboard target (200 × 150 mm; 24 corners certified to ±0.05 mm; NIST-traceable calibration); Applied Image Inc.
Data-acquisition workstation (Intel Core i7-12700K, NVIDIA RTX 3060 12 GB)	Intel; NVIDIA	i7-12700K; RTX 3060

### Experimental model and study participant details

This study recruited tennis players with provincial-level or above competition experience as subjects, all right-handed with no upper-limb injury history. A total of 15 participants (12 males and 3 females; mean age 22.3 ± 3.1 years, range 19–28) were recruited from the Beijing provincial tennis training center and the varsity team of Capital University of Physical Education and Sports. All held provincial-level second-class athlete certification or above (11 national second-class and 4 national first-class). Mean systematic training experience was 9.4 ± 2.6 years and mean weekly training volume in the three months preceding data collection was 14.2 ± 3.8 h. None reported upper-limb or lower-back injury in the 12 months preceding the experiment. The full demographic and training profile is documented in the supplemental information. This male-dominated distribution reflects the demographic composition of the recruited provincial training-centre roster and imposes a boundary condition on female-population generalisability, as flagged in the limitations of the study. Each subject received explanations of the research purpose and testing procedures and signed informed consent forms. A 15-min standardised warm-up (jogging, dynamic stretching, hitting practice) preceded formal testing. Experimental balls were new, clean, yellow ITF-certified competition balls (67 mm diameter, 57 g).

Each participant contributed approximately 12 valid trials in total (about four per stroke type for flat, topspin and slice), yielding the 178-trial dataset analyzed in the results. Since trials are drawn from the same 15 participants, the dataset has a two-level nested structure (trials nested within players), so trials cannot be assumed strictly independent at the player level. Every between-stroke-type comparison and regression model reported in the results was re-estimated under a linear mixed-effects framework (lme4 in R 4.3.2)^24^ with random intercepts at the participant level, as sensitivity analysis alongside conventional OLS and ANOVA. The participant-level ICC was 0.18 for ball speed and 0.16 for rotational angular velocity, indicating modest within-player clustering. Across all fixed-effect coefficients, mixed-effects estimates differed from OLS by less than 5% in magnitude with rank ordering and significance conclusions fully preserved, confirming the pooled trial-level analysis is not materially distorted by the nested structure. Full mixed-effects output is reported in the supplemental information.

The experimental venue was an indoor standard tennis court, shielding against natural light variations. Tan et al. emphasized that controlled lighting environments are important for visual measurement system stability.^25^ Four LED constant-current light source arrays were arranged above the hitting area, with illumination uniformity better than 95% and color temperature stable at 5600 K. The venue layout and equipment configuration are shown in Figure 10.

All procedures performed in studies involving human participants were in accordance with the 1964 Helsinki Declaration and its later amendments or comparable ethical standards. This study was approved by the Ethics Committee of the School of Recreation and Community Sport of Capital University of Physical Education and Sports (approval number: CUPES-REC-2024-037; date of approval: 15 March 2024), and written informed consent was obtained from all individual participants included in the study.

### Method details

#### System configuration and geometric calibration

Parameter configuration must balance temporal resolution, spatial resolution and system cost. Chen and Wang’s TrackNetV3 reached 97.51% tracking accuracy provided input images have sufficient spatial resolution to preserve ball feature details.^26^ This study used industrial-grade high-speed cameras with 1-inch sensor, 10 μm pixel pitch, outputting 1280 × 800 px grayscale at 2000 fps. Turner et al. confirmed in OpenCap validation that angle measurement error correlates negatively with camera resolution.^27^ This configuration ensures a 67 mm ball at 5 m has an imaging size of about 47 pixels, meeting downstream processing requirements.

Geometric calibration of binocular cameras is fundamental for three-dimensional reconstruction. Zhang’s plane calibration method^28^ was used to obtain intrinsics and distortion coefficients from a 12 × 9 checkerboard at over 30 poses. Tenzin et al. provided the theoretical framework for visual sensor calibration^29^; dynamic calibration validation for high-speed motion was added on this basis.

The exposure time was fixed at 100 μs during data collection as a deliberate compromise between motion-blur suppression and the signal-to-noise ratio required to retain seam-pattern texture under the available LED illumination. At 55 m/s, translational displacement during the 100 μs exposure is 5.5 mm in object space (≈8% of the 67 mm ball diameter, ≈4 px at the 5 m working distance), so the unblurred ball image remains a coherent disk feature whose centroid can be recovered by the motion-matched deblurring kernel described below. At the highest measured topspin rate (2836 rpm), tangential surface displacement over the same window is about 1.0 mm, well below the 5–8 mm seam-feature width and therefore preserving the texture information required by the rotation estimator. Residual translational blur is compensated by the sub-pixel ball-centre estimator described below, recovering near-pixel-level localisation precision from the raw frames.

#### Hardware synchronisation and data path

Synchronous frame triggering of the two cameras was implemented in hardware rather than software, since at 2000 fps software synchronisation cannot stay within the inter-frame jitter budget for accurate stereo reconstruction. A Xilinx Spartan-6 XC6SLX9 field-programmable gate array (FPGA) acts as the master pulse generator, distributing identical low-voltage differential signalling (LVDS) trigger pulses to both cameras via a matched-length star topology (inter-channel cable-induced delay below 1 ns) and also routing copies to the radar speed gun and the inertial reference units. The infrared light-curtain at the hitting-zone entrance provides the initiating pulse, reshaped and propagated by the FPGA. To verify the achieved synchronisation, a high-intensity LED pulse (rise time ≈80 ns) was placed in the common field of view and triggered with the cameras; across 200 events the inter-camera frame-onset offset was 67 ± 32 ns and the end-to-end trigger latency was 0.81 ± 0.04 μs (both orders of magnitude smaller than the 500 μs inter-frame period), confirming temporally coherent stereo pairs even at the upper bound of the design speed envelope.

Because 1280 × 800 px 8-bit acquisition at 2000 fps generates 2.05 GB/s per camera, full-rate capture is written to the camera’s on-board DRAM ring buffer, which sustains this rate internally, rather than streamed live to the workstation. The Camera Link Full interface (680 MB/s) is used for post-burst download of the buffered sequence: each triggered acquisition retains the ±50 ms window around impact, i.e., 201 frames ≈206 MB per camera, so the two bursts transfer in ≈0.6 s. This acquire-then-download architecture is the reason the pipeline operates in post-stroke batch mode (runtime and computational complexity) and it sets the first term of the ≈3 s coach-facing latency budget.

The hardware architecture of the ultra-high-speed photography system is shown in Figure 9, with main technical parameters listed in Table 14. To complement the technical specifications listed in Table 14, the itemised hardware bill of materials of the proposed binocular configuration is summarised in Table 11. The total cost of the prototype, including two industrial 2000 fps cameras with matched 35 mm f/1.4 lenses, the FPGA synchronous trigger generator, the LED constant-current illumination array, the data-acquisition workstation, the infrared light curtain and the calibration accessories, is approximately RMB 128,000 (≈USD 18,000). Beyond cost, the entire system can be transported and assembled by two technicians within roughly 4 h on a standard indoor court, which underpins the deployability claim used in the comparative discussion in the results.

#### Experimental protocol and data collection

The experimental design covered three stroke types: flat, topspin, and slice. For each type, every participant performed repeated returns from which the cleanly captured valid strokes (about four per stroke type per participant) were retained, defined as the ball passing through the camera field of view and landing within the valid opposite-half-court area. Tarashima et al. provided a multi-sport ball detection benchmark whose experimental design informed the camera-layout pilot phase.^30^

Data collection followed standardised procedures. Subjects stood at the baseline marker with a ball machine supplying balls at fixed parameters (120 km/h, no spin), and completed return shots of the designated type. High-speed cameras operated in external trigger mode with triggers from infrared light-curtain sensors at the hitting-zone entrance (a similar triggering mechanism was used by Nakabayashi et al.^31^). This ensures acquisition precisely covers the ±50 ms window around impact. Each stroke recorded synchronously: binocular image sequences, radar speed readings, and inertial reference data. Experimental conditions are summarised in Table 15.

Within each captured ±50 ms window the precise ball–racket impact frame was located by a two-stage detector combining velocity-reversal detection with local image-difference analysis, so that the rotation-estimation pipeline references a single contact instant rather than the coarse light-curtain timing. First, the reconstructed three-dimensional ball-centre trajectory was differentiated to obtain inter-frame velocity Vk, and the candidate impact frame k∗ was the frame at which the angle between consecutive velocity vectors exceeded 30°, an abrupt change attributable only to the racket strike. Second, a 96 × 96 px region of interest (ROI) around the racket head was extracted from a ±5-frame neighborhood of k∗, and the frame maximising the mean absolute pixel difference between consecutive ROIs was retained as the refined impact frame, giving single-frame (≈0.5 ms) precision. Across the 178 trials the two criteria agreed in 175 (98.3%); the remaining three were resolved by manual single-frame adjustment. The ±13-frame neighborhood around the refined impact frame (±6.5 ms, conservatively bracketing the 4–5 ms racket–ball contact and the subsequent elastic-rebound transient) was excluded from the rotational-attitude estimation to keep ball deformation out of the rigid-body spin calculation.

#### Image processing and parameter extraction

The image processing pipeline has four core modules: ball detection, three-dimensional reconstruction, speed calculation, and spin estimation. The overall algorithm flowchart is shown in Figure 11. Detection uses an improved YOLO architecture, with multi-scale feature fusion (after Rong^32^) fine-tuned for tennis. The MultiSenseBadminton multi-sensor dataset of Seong et al. provided useful reference for similar ball-detection tasks.^33^ The detection backbone follows YOLOv8-s with the stride-4 small-object head retained to preserve recall of the ≈47-pixel tennis ball at the 5 m working distance. A purpose-built single-class dataset (6500 images: 5200 train, 800 validation, 500 test) was drawn from pilot recordings and competition footage, manually annotated in LabelImg. Augmentation included ±30% brightness, 3 × 3–11 × 11 px motion-blur kernels, mosaic stitching and HSV jitter. Training used 640 × 640 input, batch 32, initial learning rate 0.01 with cosine annealing, 200 epochs on a single NVIDIA RTX 3060. On the held-out test set the detector reached mAP@0.5 = 97.3% and mean average precision (mAP)@0.5:0.95 = 82.8% with a per-frame miss rate below 0.5%, providing the high-recall ball-centre stream required by downstream stereo and rotation modules.

Before frames are passed to the detector and surface-texture tracker, a preprocessing step mitigates the translational motion blur from the 100 μs exposure. A coarse YOLO pass returns the ball ROI, within which non-blind Richardson–Lucy deblurring is applied with a one-dimensional kernel whose length matches the predicted inter-frame displacement and whose orientation aligns with the locally estimated velocity vector. The Kalman trajectory filter updates these kernel parameters continuously, removing manual tuning. Confining deblurring to the moving-ball ROI avoids smoothing static background structures used for calibration while still recovering seam detail. On the held-out subset with manually annotated ball centers, deblurring reduced ball-centre localisation error from ±2.5 px (raw) to ±0.8 px (deblurred) at 55 m/s, restoring sub-pixel precision for stereo triangulation.

Three-dimensional coordinate reconstruction is implemented based on binocular stereo-vision principles. Setting the left-camera coordinate system as the world coordinate system, with the ball-centre projection coordinates on the left and right camera image planes being (uL, vL) and (uR, vR) respectively, the camera baseline distance as B and the focal length as f, the ball-centre three-dimensional coordinates (X, Y, Z) can be calculated by the following equations:

(Equation 1) $$Z=\frac{Bf}{d}$$

(Equation 2) $$X=\frac{\left(u_{L}-c_{x}\right)Z}{f}$$

(Equation 3) $$Y=\frac{\left(v_{L}-c_{y}\right)Z}{f}$$

where *Z* is the depth coordinate of the ball center along the optical axis, in millimetres; *B* is the binocular camera baseline distance, in millimetres; *f* is the camera focal length, in pixels; *d* is the disparity value, in pixels; *c*_*x*_ and *c*_*y*_ are the image principal-point coordinates, in pixels; and *X* and *Y* are the ball-centre coordinates in the horizontal and vertical directions, respectively, in millimetres.

Ball speed is calculated from the ratio of the ball-centre displacement between adjacent frames to the time interval. Setting the ball-centre three-dimensional coordinates at frame k and frame k + 1 as Pk and Pk+1 respectively, with inter-frame time interval Δt, the instantaneous velocity vector Vk and its magnitude |Vk| are:

(Equation 4) $${\vec{V}}_{k}=\frac{{\vec{P}}_{k+1}-{\vec{P}}_{k}}{\Delta t}$$

(Equation 5) $$\left|{\vec{V}}_{k}\right|=\sqrt{{V_{x}}^{2}+{V_{y}}^{2}+{V_{z}}^{2}}$$

where ${\vec{V}}_{k}$ is the velocity vector at frame *k*, in m/s; Δ*t* is the inter-frame time interval, which is 0.5 ms in this system (corresponding to 2000 frames per second); and $\left|{\vec{V}}_{k}\right|$ is the scalar velocity, in m/s. Yang et al. adopted a similar velocity-calculation method in tennis-robot trajectory-prediction research and suppressed noise through Kalman filtering.^34^

#### Rotation estimation from natural seam texture

Rotational angular velocity estimation employs the ball surface texture tracking method. The white seam lines on the tennis ball surface form a characteristic figure-of-eight pattern, and the position changes of this pattern between consecutive frames reflect the ball’s rotational motion. The time-consistent ball tracking method proposed by Sato et al. provides theoretical reference for the spin estimation algorithm in this study.^35^ The orientation quaternion qk associated with each frame is not measured directly but is recovered from the image stream through a four-step procedure, which makes the link between Equations 6 and 7 and the underlying images explicit.

(i) Surface-feature initialisation and the canonical seam template. The seam pattern of an ITF-certified tennis ball is a continuous double-curved white seam ≈5–8 mm wide rather than a sharp-cornered structure, so the seam ridge does not present canonical L-shaped corners. It does, however, present a dense distribution of locally maximal second-moment-matrix eigenvalue points where the seam centreline crosses surface-curvature gradients; these behave operationally as Shi–Tomasi features under the multi-scale autocorrelation criterion.^36^ Shi–Tomasi applicability was verified empirically against four alternative detectors (Harris, FAST, ORB and a learned SuperPoint) by propagating each detector’s points with a pyramidal Lucas–Kanade tracker^37^ over 50 frames (≈25 ms) at ≈1850 rpm topspin. Shi–Tomasi yielded 38.7 stable feature points per frame with an 84.2% retention rate over that 25 ms interval, outperforming Harris (29.4 points, 76.1%), FAST (22.1, 68.4%), ORB (24.8, 71.2%) and SuperPoint (33.2, 79.6%). The Shi–Tomasi response was computed on a three-level Gaussian pyramid with a 7 × 7 integration window, points accepted only when the smaller eigenvalue exceeded 0.04 of the per-frame maximum; this concentrates features along the seam ridge where rigid-body rotation is geometrically observable. Representative feature-point visualisations under flat, topspin and slice conditions are provided in the supplemental information. On the first stable frame outside the ±13-frame impact-exclusion window, the cropped ball region is rectified to a square patch of side equal to the bounding-box diagonal and Shi–Tomasi corner detection with sub-pixel refinement is applied along the high-contrast seam ridges, yielding 30–50 surface feature points; each two-dimensional point is then back-projected onto the ideal-sphere surface using the calibrated camera intrinsics together with the reconstructed ball-centre depth, producing a set of three-dimensional surface points on the unit sphere.

The initial attitude q0 is obtained from a Procrustes alignment between these points and the canonical tennis-ball seam template stored in the calibration database. The canonical seam template is a high-resolution three-dimensional surface representation of the ITF-certified seam, constructed offline during system commissioning rather than extracted from live data. A reference ball was mounted on a precision rotary stage (±0.02°) and photographed by the binocular system at 72 azimuth orientations (5° per step, covering the full 360°) under matched diffuse illumination. The seam centreline was extracted on each image by a sub-pixel ridge detector (lateral error ≤0.3 px) and back-projected onto the unit sphere via the calibrated intrinsics and ball-centre depth. The 72 point-sets were registered through iterative closest-point alignment into a dense canonical mesh of ≈4,820 seam-centreline points, with mean RMS fit residual to the analytical ITF geometry of 0.42 mm. The mesh and its curvature descriptors are stored as a calibration artifact and reused across all stroke trials. The initial attitude q0 on any new stroke trial is recovered by Procrustes alignment between the live-frame back-projected feature points and the 36 closest candidate sub-regions of the canonical mesh, with the candidate of minimum geodesic residual retained as the initial alignment; on the present dataset, the median geodesic residual at this initialisation stage was 1.7° and exceeded 4° on fewer than 2% of trials. The full template-construction protocol and the corresponding calibration artifacts are documented in Data S1.

(ii) Inter-frame association: feature points are propagated by a pyramidal Lucas–Kanade tracker; trajectories violating the binocular epipolar constraint or with normalised cross-correlation below 0.7 are rejected through random sample consensus (RANSAC), and lost points are replenished by re-detection so that the active set stays above the six-point minimum needed for a well-conditioned rigid-body fit. (iii) Quaternion update: surviving correspondences are passed to Horn’s closed-form unit-quaternion estimator,^38^ which returns the rotation increment Δq minimising squared geodesic error; the per-frame attitude is accumulated as qk+1 = Δq ⊗ qk. (iv) Temporal smoothing: a third-order Savitzky–Golay filter with a 7-frame window suppresses jitter without blurring genuine rotation increments. The 7-frame window corresponds to 3.5 ms at 2000 fps and was selected on a held-out subset by minimising the residual between the smoothed rotation-increment sequence and the corresponding gyroscope reference, balancing jitter suppression against attenuation of physically genuine rotational transients; windows of 5 and 9 frames increased that residual by 12% and 9% respectively. The ±13-frame window around contact is excluded because peak ball compression reaches ≈25% of the ball diameter and violates the rigid-body assumption of Equations 6 and 7; restricting to steady-state pre- and post-contact frames isolates rotation estimation from the deformation transient. Setting the attitude quaternion at frame k as qk = (q0, q1, q2, q3) and at frame k + 1 as qk+1, the rotation increment quaternion Δq and the rotational angular velocity ω can be calculated by the following equations:

(Equation 6) $$\Delta q=q_{k+1}⊗{q_{k}}^{-1}$$

(Equation 7) $$\omega =\frac{2\;\arccos\left({\Delta q}_{0}\right)}{\Delta t}$$

where ⊗ represents quaternion multiplication; ${q_{k}}^{-1}$ is the inverse of the attitude quaternion; Δ*q*_0_ is the scalar part of the rotation-increment quaternion; and *ω* is the rotational angular velocity, in radians per second. The ball-surface-texture-tracking principle is shown schematically in Figure 12.

### Quantification and statistical analysis

Statistical details for each experiment (including the test used, the exact value of n, what n represents, and the dispersion and precision measures) are reported in the corresponding figure legends, table footnotes and Results subsections. Unless otherwise stated, n denotes the number of valid stroke trials (*n* = 178; 59 flat, 62 topspin, 57 slice) and summary data are reported as mean ± standard deviation (SD). In Figure 3, asterisks denote post-hoc pairwise comparisons following a one-way ANOVA with Bonferroni correction, with ∗∗∗ indicating *p* < 0.001. In the Bland–Altman plot (Figure 5) the solid line denotes the mean bias and the dashed lines the 95% limits of agreement (mean bias ±1.96 SD). Correlation coefficients in Figures 4 and 7 are Pearson’s r with the associated *p* value. Statistical significance was defined at α = 0.05 (two-tailed). For the post-hoc pairwise comparisons following ANOVA, family-wise error was controlled by the Bonferroni method; regression coefficient *p* values are reported without further multiplicity correction, since each coefficient tests a pre-specified hypothesis derived from the theoretical framework of the introduction. Statistical analysis was performed using SPSS 26.0 and cross-checked under a linear mixed-effects framework using the lme4 package in R 4.3.2.^24^

Error sources of the measurement system include three levels: image acquisition error, feature extraction error, and three-dimensional reconstruction error. Phomsoupha et al. cross-validated Doppler radar and high-speed camera systems in badminton speed measurement reliability research, with intraclass correlation coefficients reaching 0.88–0.94.^39^ This study referenced this validation framework to design accuracy assessment experiments. The ball speed inversion method using racket vibration signals adopted by the SmartDampener open-source tennis analysis platform provides an additional reference data source for this study.^40^

#### Independent three-dimensional reconstruction calibration

To independently verify three-dimensional reconstruction accuracy without circular back-calculation from ball-speed RMSE, two complementary calibration experiments were executed before the formal stroke-data campaign. The first was a static three-dimensional reconstruction calibration: a precision three-axis checkerboard with 24 corners certified at ±0.05 mm was placed at nine prescribed poses in the central 1.5 × 1.0 × 1.0 m working volume (space diagonal 2061.6 mm), and the per-axis deviation across all 216 corner observations (24 corners × 9 poses) was reported as the static reconstruction error. The second was a dynamic reconstruction calibration: a 67 mm calibration sphere matching the tennis-ball surface finish was mounted on a precision servo-driven linear stage (0.1 μm encoder, ±20 μm repeatability) and driven through the working volume at constant speeds of 5, 10, 15 and 20 m/s, with 20 repetitions per speed (80 dynamic trials total). The static experiment isolates geometric calibration residual and disparity-to-depth conversion error; the dynamic experiment additionally probes systematic bias from inter-frame tracking and Kalman smoothing. Per-axis RMSE values are reported in Table 2, and the combined three-dimensional position RMSE of each row is the root sum of squares of its three per-axis values, so that every entry can be independently recomputed. Results provide direct mechanism-level evidence for hypothesis H1, replacing the indirect ball-speed back-calculation used previously.

#### Method-comparison framework

Method comparison between the proposed system and reference instruments (radar speed gun, smart-ball gyroscope, blinded human-annotation reference) is reported within a Bland–Altman agreement framework^41^ as the primary metric, with the paired-difference *t* test, Cohen’s d_z, and Bayesian paired-samples *t* test^42^ as supporting indicators rather than principal evidence of equivalence. This framework was adopted because a non-significant paired *t* test alone cannot be interpreted as evidence of equivalence, while Bland–Altman analysis directly visualises the joint behavior of mean bias and dispersion. For each comparison the following quantities are reported in the results: mean bias, SD of paired differences, 95% limits of agreement, 95% CI of the mean bias, and a pre-defined practically acceptable equivalence margin. The margin was set to ±2.5 km/h for ball speed, derived from typical inter-trial stroke variability (≈2 km/h) plus the radar reference half-width (±0.16 km/h) and a small allowance for aerodynamic asymmetry, and to ±5% of the reference value for rotational angular velocity, matching the H2 threshold. Comparisons against a single instrument’s nominal accuracy are made cautiously: when the upper bound of the bias 95% CI exceeds the nominal reference accuracy, agreement is described as falling within the pre-defined margin rather than entirely within the reference instrument’s error band.

Two conventions within this framework should be stated explicitly, because they mean that the reported limits of agreement and the reported confidence interval of the bias rest on different standard errors. The 95% limits of agreement are computed as mean bias ±1.96 × SD of the raw paired differences, as the Bland–Altman method prescribes, because the limits are intended to describe the dispersion of individual paired differences and must not be narrowed by the sample size. The 95% CI of the mean bias, the supporting paired t statistic and Cohen’s d_z are, by contrast, inferential statements about the mean and are therefore corrected for the two-level nested structure of the data (trials nested within participants) using a cluster-robust standard error at the participant level. For the camera–radar comparison the mean cluster size is 11.9 trials per participant and the participant-level ICC of the paired differences is ≈ 0.14, giving a design effect of ≈2.52 and inflating the naive standard error by a factor of ≈1.59; the reported CI of [−0.02, +0.90] km/h, t(177) = 1.87 and d_z = 0.14 are the cluster-corrected values, whereas the reported limits of agreement of [−3.44, +4.32] km/h follow directly from the 1.98 km/h raw paired-difference SD of Table 3. Applying the cluster correction is the conservative choice and is consistent with the mixed-effects sensitivity analysis applied to every other inferential model in this study.

For the two rotational-angular-velocity comparisons (Tables 7 and 8), the mean absolute deviation and the mean absolute deviation rate are reported as the accuracy indicators against which the ±5% H2 threshold is evaluated, while the mean signed difference and the SD of signed differences are reported separately as the inputs to the paired *t* test and the RMSE. These are distinct quantities: the absolute deviation measures typical error magnitude irrespective of direction, whereas the signed difference measures systematic offset. Reporting both makes each row of Tables 7 and 8 independently recomputable.

#### Accuracy metrics

Ball speed measurement accuracy is further quantified using root-mean-square error (RMSE) and mean absolute percentage error (MAPE), calculated as follows:

(Equation 8) $$\text{RMSE}=\sqrt{\frac{1}{n}\sum_{i=1}^{n}{\left(v_{i}-{\hat{v}}_{i}\right)}^{2}}$$

(Equation 9) $$\text{MAPE}=\frac{100\%}{n}\sum_{i=1}^{n}\frac{\left|v_{i}-{\hat{v}}_{i}\right|}{{\hat{v}}_{i}}$$

where *n* is the number of measurement samples; *v*_*i*_ is the system output value for the *i*-th measurement; ${\hat{v}}_{i}$ is the reference-system reading for the *i*-th measurement; RMSE carries the units of the measured quantity; and MAPE is a dimensionless percentage. Because MAPE is the arithmetic mean of per-trial relative errors, the all-sample MAPE of Table 3 equals the sample-size-weighted mean of the three per-stroke-type values.

#### Mixed-effects sensitivity analysis

Multiple linear regression analysis was used to test the coupling relationship between ball speed and rotational angular velocity in hypothesis H4, with independent variables including stroke type, racket face angle, and swing speed, and dependent variable being ball rotational angular velocity. Because the 178 stroke trials are nested within 15 participants, every regression and between-group comparison reported in the results was additionally re-estimated under a two-level linear mixed-effects model with a random intercept at the participant level, as a sensitivity analysis. Variance components, the participant-level intraclass correlation coefficient and the resulting fixed-effect coefficients are reported alongside the conventional ordinary-least-squares estimates, with the full mixed-effects output documented in the supplemental information. If the mixed-effects estimates differ from the OLS estimates by more than 5% in magnitude for any predictor, or if the rank ordering of standardised contributions changes, this is flagged explicitly in the Results; on the present dataset no such flag was triggered.

### Additional resources

This study was not registered in any public trial or study registry, and it is not associated with any consortium, portal or dedicated website. The canonical seam-template artifacts, their curvature descriptors, the rotary-stage image set and the construction protocol required to rebuild the rotation estimator from scratch are provided as Data S1. All remaining datasets, the annotated detection dataset and the full processing code are available from the lead contact as set out in the data and code availability statement.

## Supplemental information

Document S1. Figures S1 and S2 and Tables S1 and S2

Data S1. Canonical seam-template construction protocol and the corresponding calibration artifacts, related to STAR Methods Provided as a compressed archive containing the dense canonical seam mesh, its curvature descriptors, the rotary-stage image set, and the construction protocol
