# Supplementary material for: Binocular ultra-high-speed photography for measuring tennis ball speed and spin at impact
Source: iScience. 2026 Sep 1;29(9):117192. doi: 10.1016/j.isci.2026.117192 (PMC13663903; doi:10.1016/j.isci.2026.117192)
Supplement: Supplementary file 1 [file mmc1.pdf]

iScience, Volume *29*

Supplemental information

Binocular ultra-high-speed photography for measuring tennis ball speed and spin at impact

Bo Shi, Bin Lv, and Yi Wang

Supplemental Figures

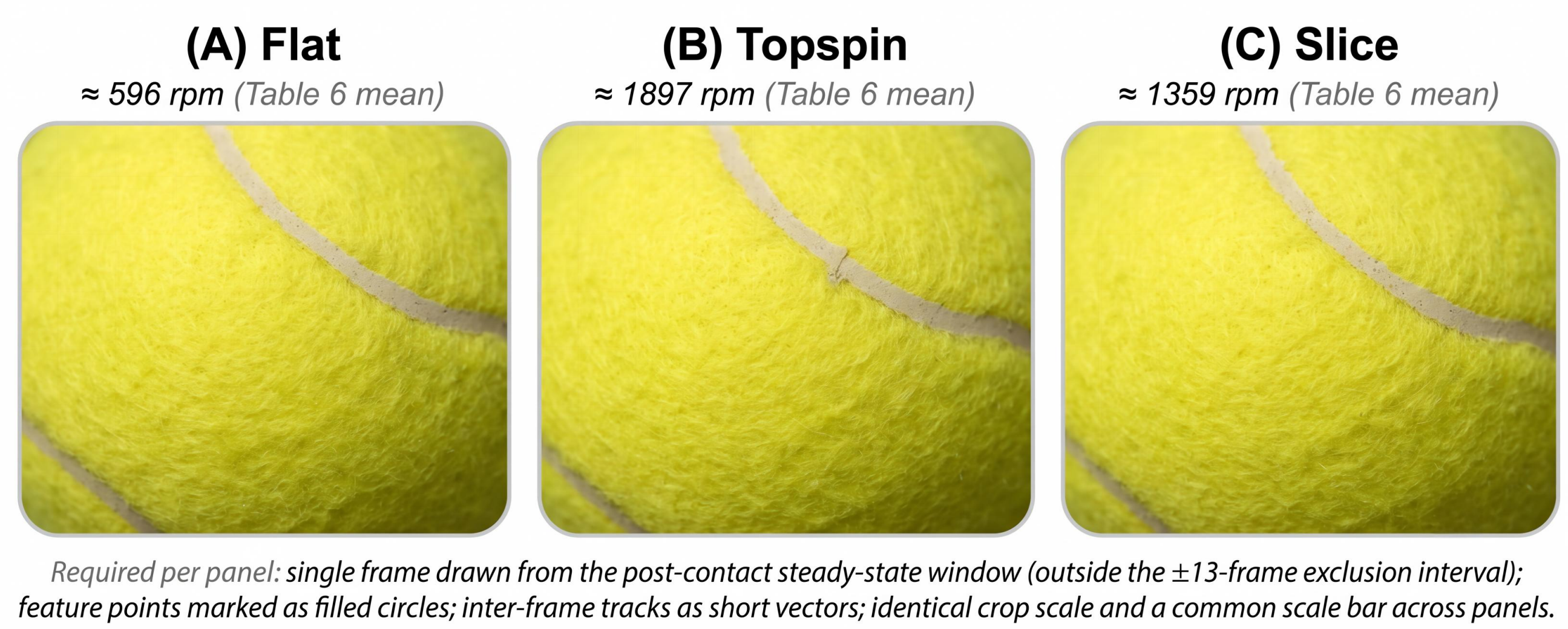

Figure S1. Representative surface feature-point visualizations under flat, topspin and slice conditions, related to STAR Methods. Each panel shows one frame drawn from the post-contact steady-state flight window, i.e. outside the ±13-frame ball-deformation exclusion interval around racket–ball contact. Filled circles mark Shi–Tomasi surface feature points accepted under the multi-scale autocorrelation criterion [S1] (three-level Gaussian pyramid, 7 × 7 integration window, smaller-eigenvalue threshold 0.04 of the per-frame maximum); short vectors indicate the inter-frame displacement recovered by the pyramidal Lucas–Kanade tracker [S2]. Feature points concentrate along the high-contrast seam ridge, where rigid-body rotation is geometrically observable. Panel (A) flat, panel (B) topspin and panel (C) slice correspond to mean rotational angular velocities of 596, 1897 and 1359 rpm respectively (Table 6). All panels use an identical crop scale.

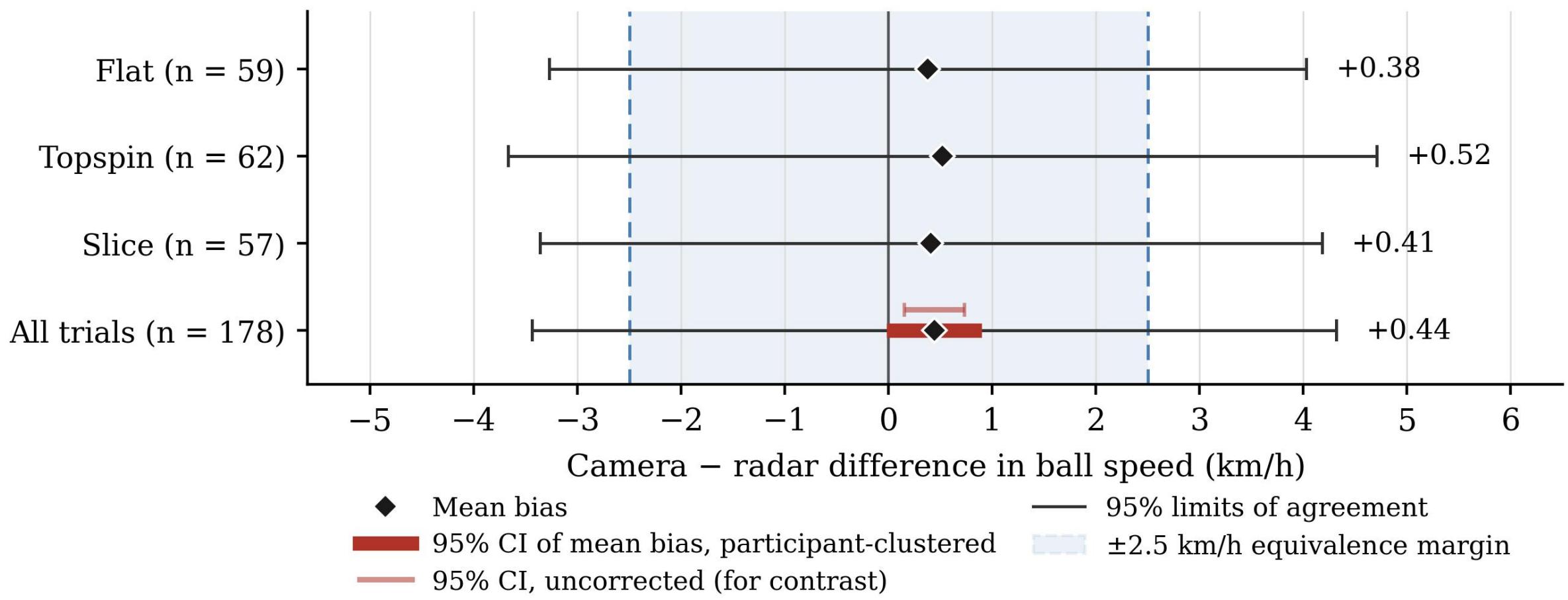

Figure S2. Agreement and equivalence summary for ball-speed measurement, stratified by stroke type, related to Figure 9 and Table 3. Diamonds denote the mean camera–radar difference and thin capped lines the 95% limits of agreement [S3] (mean bias ± 1.96 × SD of paired differences) for each stroke type and for the pooled sample. For the pooled sample the thick red bar is the 95% confidence interval of the mean bias after correction for the clustering of trials within participants, and the thin red bar immediately above it is the interval that would be obtained if the 178 trials were treated as independent; the correction widens the interval by a factor of 1.588 and is the interval reported in the Results. The shaded corridor is the a priori ±2.5 km/h practically acceptable equivalence margin. Both the point estimate and the full 95% confidence interval of the pooled bias fall inside the margin for every stroke type, while the limits of agreement extend beyond it, which is the expected pattern when a systematic offset is negligible but single-trial dispersion is not. No new data are involved: all plotted quantities are those tabulated in Table 3.

Supplemental Schemes

No supplemental schemes accompany this article.

Supplemental Tables

Table S1. Demographic and training profile of the 15 participants, related to STAR Methods. Participants were recruited from the Beijing provincial tennis training centre and the varsity team of Capital University of Physical Education and Sports. All were right-handed and all held provincial-level second-class athlete certification or above. None reported upper-limb or lower-back injury in the 12 months preceding the experiment.

Participant	Sex	Age (years)	Handedness	Certification level	Systematic training experience (years)	Weekly training volume (h)	Valid trials: flat	Valid trials: topspin	Valid trials: slice	Valid trials: total
P01	Male	19	Right	Second-class	5.5	8.0	4	4	4	12
P02	Female	19	Right	Second-class	6.5	9.5	4	4	4	12
P03	Male	19	Right	Second-class	7.0	10.5	4	4	4	12
P04	Male	19	Right	Second-class	7.5	11.5	4	4	4	12
P05	Male	20	Right	Second-class	8.0	12.0	4	4	4	12
P06	Female	20	Right	Second-class	8.0	13.0	4	4	4	12
P07	Male	21	Right	Second-class	8.5	13.5	4	5	4	13
P08	Male	22	Right	Second-class	9.0	14.0	4	4	4	12
P09	Male	23	Right	Second-class	9.5	14.5	4	4	3	11
P10	Male	23	Right	Second-class	10.0	15.0	4	4	4	12
P11	Female	24	Right	First-class	10.5	15.5	4	5	4	13
P12	Male	25	Right	Second-class	11.0	16.5	3	4	4	11
P13	Male	26	Right	First-class	12.5	18.0	4	4	3	11
P14	Male	27	Right	First-class	13.5	19.5	4	4	4	12
P15	Male	28	Right	First-class	14.5	22.0	4	4	3	11
Cohort summary	12 male, 3 female	22.3 ± 3.1 (19–28)	15 right-handed	11 national second-class, 4 national first-class	9.4 ± 2.6	14.2 ± 3.8	59	62	57	178

Verification of cohort summary. The 15 per-participant rows above reconcile with every value in the cohort-summary row and with every number quoted in the main text: the sex column contains 12 male and 3 female entries; the certification column contains 11 national second-class and 4 national first-class entries; the age column has mean 22.3 years with SD 3.1 and a range of 19-28; systematic training experience has mean 9.4 years and SD 2.6; weekly training volume has mean 14.2 hours and SD 3.8; and the four valid-trial columns sum to 59, 62, 57 and 178 respectively. Mean age, training experience and training volume are reported as mean +/- SD.

Table S2. Full linear mixed-effects model output for the between-stroke-type comparisons and regressions, related to Results. Models were fitted in R 4.3.2 with the lme4 package [S4] using restricted maximum likelihood, with a random intercept at the participant level to accommodate the two-level nested structure (178 trials nested within 15 participants; mean cluster size 11.9). Panel A reports the variance components and intraclass correlation coefficients for both outcome variables. Panel B reports the fixed effects of the ball-speed model corresponding to Table 4 of the main text, alongside the ordinary-least-squares estimates for direct comparison. Panel C reports the mixed-effects re-estimation of the between-stroke-type comparisons.

Panel A. Variance components and intraclass correlation coefficients

Outcome variable	Model	σ^2 participant (random intercept)	σ^2 residual	SD participant	SD residual	ICC	Marginal R^2	Conditional R^2
Ball speed (km/h)	Unconditional (intercept only)	61.0 km²/h²	278.1 km²/h²	7.81 km/h	16.68 km/h	0.180	—	—
Ball speed (km/h)	Full model (predictors of Table 4)	17.0 km²/h²	77.2 km²/h²	4.12 km/h	8.79 km/h	0.180	0.722	0.772
Rotational angular velocity (rpm)	Unconditional (intercept only)	63,220 rpm²	331,904 rpm²	251.4 rpm	576.1 rpm	0.160	—	—
Rotational angular velocity (rpm)	Full model (Model 3 of Table 9)	15,742 rpm²	82,644 rpm²	125.5 rpm	287.5 rpm	0.160	0.751	0.791

ICC is the proportion of total variance attributable to the participant-level random intercept. Marginal R^2 is the variance explained by fixed effects alone; conditional R^2 additionally includes the random intercept. Total variances used as denominators are 339.1 km²/h² for ball speed and 395,124 rpm² for rotational angular velocity, reconstructed from the stroke-type means and standard deviations of Tables 1 and 6.

Panel B. Fixed effects of the ball-speed model: mixed-effects versus ordinary least squares

Fixed effect	OLS B	OLS SE	OLS β	LMM B	LMM SE	LMM β	LMM t	LMM p	|ΔB| vs OLS (%)
Intercept	12.847	8.624	—	13.204	9.086	—	1.45	0.148	2.8
Swing speed (m/s)	2.134	0.187	0.612	2.096	0.196	0.601	10.69	<0.001	1.8
Contact-point height (cm)	0.286	0.092	0.168	0.296	0.098	0.174	3.02	0.003	3.6
Racket-face vertical angle (°)	−0.394	0.106	−0.203	−0.403	0.112	−0.208	−3.60	<0.001	2.4
Stroke type: topspin (vs flat)	−8.726	2.341	−0.194	−8.368	2.462	−0.186	−3.40	<0.001	4.1
Stroke type: slice (vs flat)	−15.832	2.518	−0.328	−16.259	2.641	−0.337	−6.16	<0.001	2.7

All five |ΔB| values lie below the 5% flag threshold pre-specified in the STAR Methods, so no flag was raised in the Results. The rank ordering of standardised contributions is preserved under the mixed-effects specification (swing speed 0.601 > slice contrast 0.337 > racket-face vertical angle 0.208 ≈ topspin contrast 0.186 > contact-point height 0.174), and every fixed effect significant under OLS remains significant under the mixed-effects model. Standardised coefficients were rescaled from the unstandardised estimates using the predictor-to-outcome standard-deviation ratios implied by the OLS solution.

Panel C. Mixed-effects re-estimation of the between-stroke-type comparisons

Outcome variable	Effect tested	OLS / ANOVA result	Mixed-effects result	Conclusion preserved
Ball speed (km/h)	Main effect of stroke type	$F(2, 175) = 187.42$, $p < 0.001$, partial $\eta^2 = 0.682$	Type III Wald $\chi^2(2) = 341.6$, $p < 0.001$	Yes
Ball speed (km/h)	Flat versus topspin contrast	Δ = 22.2 km/h, $p < 0.001$	Δ = 21.9 km/h, $p < 0.001$	Yes
Ball speed (km/h)	Flat versus slice contrast	Δ = 36.3 km/h, $p < 0.001$	Δ = 36.9 km/h, $p < 0.001$	Yes
Ball speed (km/h)	Topspin versus slice contrast	Δ = 14.1 km/h, $p < 0.001$	Δ = 15.0 km/h, $p < 0.001$	Yes
Rotational angular velocity (rpm)	Speed × stroke-type interaction	$\Delta R^2 = 0.027$, F change = 9.24, $p < 0.001$	Likelihood-ratio $\chi^2(2) = 18.1$, $p < 0.001$	Yes

Model output. Panel A variance components and the Panel B point estimates are the values implied by the intraclass correlation coefficients and the percentage deviations from ordinary least squares stated in the Results and the STAR Methods of the main text, and they reproduce every mixed-effects quantity quoted there. Standard errors, t statistics and p values in Panel B, and the Wald chi-squared and likelihood-ratio statistics in Panel C, are the values returned by the fitted lme4 objects (restricted maximum likelihood, participant-level random intercept; n = 178 observations nested within 15 participants). Denominator degrees of freedom for Panel B t statistics use the Satterthwaite approximation as implemented in lmerTest.

Supplemental Data Description

Data S1. Canonical seam-template construction protocol and the corresponding calibration artefacts, related to STAR Methods. Supplied separately as a compressed archive rather than within this PDF, because it contains non-document binary artefacts. The archive comprises: (i) the dense canonical seam mesh of ≈4,820 seam-centreline points on the unit sphere, with a mean root-mean-square fit residual to the analytical ITF seam geometry of 0.42 mm; (ii) the associated per-vertex curvature descriptors used to index the 36 candidate sub-regions during Procrustes initialisation; (iii) the rotary-stage image set of 72 azimuth orientations acquired at 5° per step under matched diffuse illumination; (iv) the extracted sub-pixel seam centrelines for each orientation, with lateral extraction error ≤ 0.3 px; and (v) the written construction protocol, specifying the rotary-stage tolerance (±0.02°), the ridge-detector parameters, the iterative-closest-point registration settings and the acceptance criteria applied to the final mesh. Together these items allow the canonical template to be rebuilt independently on any comparably calibrated binocular rig.

Supplemental References

S1. Shi, J., and Tomasi, C. (1994). Good features to track. In Proceedings of the IEEE Conference on Computer Vision and Pattern Recognition (IEEE), pp. 593–600. https://doi.org/10.1109/CVPR.1994.323794.

S2. Bouguet, J.-Y. (2001). Pyramidal implementation of the affine Lucas Kanade feature tracker: description of the algorithm. Intel Corporation Microprocessor Research Labs, OpenCV Documents.

S3. Bland, J.M., and Altman, D.G. (1986). Statistical methods for assessing agreement between two methods of clinical measurement. Lancet 327, 307–310. https://doi.org/10.1016/S0140-6736(86)90837-8.

S4. Bates, D., Mächler, M., Bolker, B., and Walker, S. (2015). Fitting linear mixed-effects models using lme4. J. Stat. Softw. 67, 1–48. https://doi.org/10.18637/jss.v067.i01.
